# Interactions between infant characteristics and parenting factors rarely replicate across cohorts and developmental domains

**DOI:** 10.1111/jcpp.14149

**Published:** 2025-03-10

**Authors:** Robert Eves, Finiki Nearchou, Dieter Wolke, Michael Pluess, Sakari Lemola

**Affiliations:** ^1^ Department of Psychology Bielefeld University Bielefeld Germany; ^2^ Department of Psychology, Lifespan Health and Wellbeing Group University of Warwick Coventry UK; ^3^ School of Psychology University College Dublin Dublin Ireland; ^4^ Department of Biological and Experimental Psychology Queen Mary University of London London UK; ^5^ School of Psychology University of Surrey Guildford UK

**Keywords:** Interaction, moderation, birthweight, temperament, vulnerability, susceptibility, sensitivity

## Abstract

**Background:**

Whether, and how, infant characteristics and parenting quality interact is one of developmental psychology's key questions. However, whether specific interaction patterns replicate across cohorts or developmental outcomes is largely unknown. This study investigates whether infant characteristics and parenting quality are independent predictors (additive effects) of child outcomes or interact such that certain infants particularly suffer from poor parenting (diathesis stress), particularly benefit from good parenting (vantage sensitivity) or both (differential susceptibility).

**Methods:**

Individual participant data from over 30,000 children from four prospective cohorts were pooled. Using a competitive‐confirmatory approach of model evaluation, 16 possible permutations of infant characteristics (temperament and birthweight), parenting (maternal‐reported stimulating and sensitive parenting) and later developmental outcomes (fluid and crystalised intelligence, internalising and externalising behaviour) were tested. The robustness of results was evaluated by subsequently varying analytic methods, using alternative parenting measures including observer reports and excluding covariates.

**Results:**

AIC values in 10/16 analyses indicated infant characteristics acted independently of maternal‐reported parenting for predicting developmental outcomes. Interaction patterns indicating diathesis stress (4/16), vantage sensitivity (2/16) or differential susceptibility (0/16) were rare or absent. However, diathesis‐stress patterns were frequently found regarding birthweight and internalising behaviours, which were largely robust to methodological changes.

**Conclusions:**

Developmental outcomes are more consistently explained by additive effects rather than by interaction effects.

## Introduction

**Figure 1:**
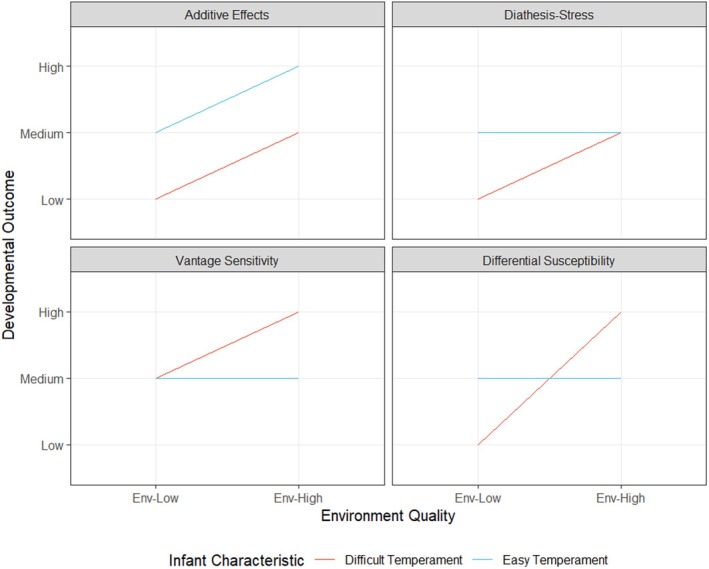
Theoretical relationships between infant characteristics (example of infant temperament shown), environmental factors and developmental outcomes. Adapted from similar figures in Widaman et al. (2012) and Jolicouer et al. (2020)

A fundamental question concerning human development is how, and to what extent, individual characteristics and environmental factors influence developmental outcomes together. Specifically, competing theoretical models of person‐environment interplay have been proposed, with varying degrees of empirical support. The most basic model indicates that individual characteristics (i.e. an infant's temperament), and their environment (i.e. the parenting they receive), ‘independently’ or ‘additively’ impact developmental outcomes. This ‘additive’ or ‘main effects model’ is contrasted by three possible interactive models (Figure 1). First, the diathesis‐stress model postulates that certain ‘risk’ characteristics, such as a difficult infant temperament, make individuals disproportionately vulnerable to the negative effects of poorer parenting or other adverse environments (Belsky & Pluess, 2009; Colodro‐Conde et al., 2018). Furthermore, a diathesis‐stress pattern indicates that infants with difficult or easy temperaments perform equivalently on developmental outcomes when both are raised in similarly low‐risk environments (i.e. receiving high‐quality parenting) (Widaman et al., 2012). Finally, a strict diathesis‐stress pattern suggests that individuals without a risk characteristic are largely unaffected by environmental quality.

A further interaction pattern is vantage sensitivity. It stipulates that some individuals disproportionately benefit from high‐quality environments due to characteristics that increase their sensitivity to the positive effects of supportive environments (Pluess & Belsky, 2013). For example, infants with difficult temperaments may instead benefit more from stimulating parenting resulting in superior developmental outcomes than infants with easier temperaments (Ng‐Knight & Schoon, 2021). Crucially, vantage sensitivity postulates that individuals with vantage sensitivity characteristics (e.g. difficult temperament) may not differ from vantage‐resistant individuals (e.g. easy temperament) when both are raised in lower quality environments, with vantage‐resistant individuals largely unaffected by environmental quality (Pluess & Belsky, 2013). The last interaction pattern, known as differential susceptibility, stipulates that characteristics such as a difficult temperament are indicators of developmental plasticity ‘for better and for worse’ (Belsky, Bakermans‐Kranenburg, & van IJzendoorn, 2007). Thus, infants with difficult temperaments may disproportionately suffer when in low‐quality environments but also disproportionately benefit from high‐quality environments, more so than individuals without the specific susceptibility characteristic (Belsky & van IJzendoorn, 2017).

Numerous infant characteristics have been considered to determine whether additive models, diathesis stress, vantage sensitivity or differential susceptibility most accurately or most often reflect developmental processes. Most prominently, infant temperament (Slagt, Dubas, Deković, & van Aken, 2016) and to a lesser degree, perinatal characteristics (e.g. low birthweight (Jaekel, Pluess, Belsky, & Wolke, 2015), small for gestational age (Nichols, Jaekel, Bartmann, & Wolke, 2020), preterm birth (Gould et al., 2022)) have been considered as characteristics supporting one of the aforementioned theoretical models. As well as an additive effect, a difficult temperament has been hypothesised as a marker of vulnerability (Rioux, Castellanos‐Ryan, Parent, & Séguin, 2016), sensitivity (Pluess & Belsky, 2013) or susceptibility (Belsky et al., 2007). Regarding susceptibility, infants with difficult temperaments may have highly susceptible nervous systems, resulting in individuals being more strongly influenced by their environment, for better and for worse (Belsky & Pluess, 2009). This has been subsequently supported by a meta‐regression (Slagt et al., 2016). However, other studies find additive effects (Licata‐Dandel, Wenzel, Kristen‐Antonow, & Sodian, 2021), diathesis stress (Gobeil‐Bourdeau, Lemelin, Letarte, & Laurent, 2022) or vantage sensitivity (Ng‐Knight & Schoon, 2021). Certain aspects of a difficult temperament, including the negative affect to novelty, were found to be advantageous in positive environments but not deleterious in negative environments, indicating vantage sensitivity (Ng‐Knight & Schoon, 2021).

As well as temperament, birthweight is frequently considered an important infant characteristic for later outcomes. When solely considering birthweight, many studies report positive associations with developmental outcomes (Gu et al., 2017; Mathewson et al., 2017). However, when in low‐quality environments, multiple studies report lower birthweight infants performing disproportionately worse, indicating diathesis stress (Camerota, Willoughby, Cox, & Greenberg, 2015; Jaekel et al., 2015; Wolke, Jaekel, Hall, & Baumann, 2013; Wu & Chiang, 2016). Finally, differential susceptibility has been theorised as lower birthweight may indicate a non‐optimal prenatal environment, resulting in ‘prenatal programming of postnatal plasticity’, resulting in increased sensitivity to the anticipated postnatal environment (Pluess & Belsky, 2011; Wu & Chiang, 2016).

In this domain, parenting factors are the most commonly studied environmental factors (Belsky et al., 2007). Specifically, more cognitively stimulating and sensitive parenting have been consistently positively associated with child development (Lahey et al., 2008; Nichols et al., 2020). However, it is unclear if parenting factors have additive effects or whether they interact with infant characteristics (Bradley et al., 1993; Gould et al., 2022; Jaekel et al., 2015; Nichols et al., 2019; Slagt et al., 2016). Additionally, studies investigating the person‐environment interplay have considered numerous developmental outcomes. Relative to other developmental outcomes, researchers have suggested that differential susceptibility may be less applicable to cognitive outcomes (Gueron‐Sela, Atzaba‐Poria, Meiri, & Marks, 2015; Jaekel et al., 2015; Roisman et al., 2012), although this has been disputed (Slagt et al., 2016). Moreover, it is debated whether diathesis‐stress, vantage‐sensitivity and differential‐susceptibility effects are ‘domain general’ or ‘domain specific’ (Belsky et al., 2007; Belsky, Zhang, & Sayler, 2021; Loginova & Slobodskaya, 2021). Environmental sensitivity may reflect a general trait and thus findings should be robust to which environmental factors or developmental outcomes are considered, or how factors are measured (e.g. maternal report or observer report) (Jaekel et al., 2021; Roisman et al., 2012). However, there is little empirical research into whether effects are domain‐general and whether methodological changes influence findings (Belsky et al., 2021). Overall, why research focuses on specific combinations of individual characteristics, environmental factors and developmental outcomes is rarely explained.

In this study, we investigate how often each model of person‐environment interplay (additive effects, diathesis stress, vantage sensitivity and differential susceptibility) is supported. Using individual participant data (IPD) from four cohorts, individual characteristics (infant temperament and birthweight) and environmental factors (cognitively stimulating parenting and sensitive parenting) are considered for predicting key developmental outcomes (fluid and crystallised intelligence, internalising and externalising behaviours). Relatedly, we determine how often effects replicate across cohorts and if the methodology is changed. Relative to aggregate meta‐analysis and meta‐regression, harmonising IPD from multiple cohorts results in being able to run equivalent analyses and adjust for important covariates (Tierney et al., 2015). Furthermore, methodological decisions such as the inclusion of covariates, using parent or observer‐reported variables or testing for nonlinear associations may influence which model of person‐environment interplay is supported. Overall, we test each model of person‐environment interplay for its specificity vs generality across developmental domains, its universality across cohorts and its robustness to methodological changes.

## Methods

### Cohorts

Open‐access data from four, large, longitudinal cohorts from high‐income, English speaking countries were used: the Millennium Cohort Study (MCS; the United Kingdom of Great Britain and Northern Ireland) (Connelly & Platt, 2014), The Longitudinal Study of Australian Children (LSAC; the Commonwealth of Australia) (Soloff, Lawrence, & Johnstone, 2005), the National Longitudinal Survey of Youth 1979 – children and young adults (NLSY79 – The United States of America) (Rothstein, Carr, & Cooksey, 2019) and the Growing up In Ireland study (GUI; the Republic of Ireland) (Williams, Murray, McCrory, & McNally, 2013). Further information on these samples has been reported elsewhere, but is outlined in Table 1. To improve cohort comparability, participants were included if their infant temperament could be measured. Thus, LSAC and GUI participants recruited in childhood were excluded while the NLSY79 did not assess temperament before 1986 or after 2000 (Chen, 2012).

**Table 1:** Demographics and measures used for each cohort

	GUI (Ireland)	LSAC (Australia)	MCS (UK)	NLSY79 (USA)
	(*N* = 8,703)	(*N* = 4,316)	(*N* = 13,124)	(*N* = 4,968)
Cohort birth year range	2007–2008	2003–2004	2000–2002	1984–2000
Temperament (Z)^a^	Infant Characteristics Questionnaire (9 months)	The Short Temperament Scale (0–12 months)	Carey Infant Temperament Scale (9 months)	The NLSY79's Temperament Scale (0–24 months)
Median [Min, Max]	0.173 [−4.86, 1.78]	0.0575 [−3.57, 2.47]	0.141 [−4.41, 2.58]	−0.0723 [−3.64, 2.26]
Missing	19 (0.2%)	491 (11.4%)	346 (2.6%)	807 (16.2%)
Birthweight (*Z*)	Parent‐reported (9 months)	Parent‐reported (0–12 months)	Parent‐reported (9 months)	Parent‐reported (0–24 months)
Median [Min, Max]	0.0335 [−3.70, 2.08]	0.0789 [−5.32, 3.57]	0.0630 [−4.91, 5.76]	0.0609 [−5.35, 6.84]
Missing	96 (1.1%)	17 (0.4%)	8 (0.1%)	0 (0%)
Stimulating parenting – Maternal‐reported (*Z*)	PCA Created Scale‐7 Items (3 years)	Indoor and Outdoor Activities Index (3 years)	PCA Created Scale‐ 6 Items (3 years)	HOME‐Stimulating (3 years)
Median [Min, Max]	0.0264 [−3.33, 1.69]	0.0411 [−3.37, 2.73]	0.0652 [−2.60, 1.84]	0.376 [−5.27, 1.57]
Missing	4 (0.0%)	0 (0%)	1 (0.0%)	0 (0%)
Sensitive parenting – Maternal reported (*Z*)	LSAC Parenting Style Questionnaire (3 years)	LSAC Parenting Style Questionnaire (3 years)	Straus Conflicts and Pianta Scales (3 years)	HOME‐Sensitive (3 years)
Median [Min, Max]	0.142 [−5.15, 1.84]	0.132 [−5.32, 1.75]	0.0725 [−4.56, 2.11]	0.0749 [−3.61, 1.45]
Missing	0 (0%)	948 (22.0%)	3,345 (25.5%)	0 (0%)
Internalising behaviours (*Z*)^a^	SDQ‐emotional and peer problems (5 years)	SDQ‐emotional and peer problems (5 years)	SDQ‐emotional and peer problems (5 years)	BPI‐Internalising problems (5 years)
Median [Min, Max]	0.197 [−5.21, 1.03]	0.328 [−5.01, 1.15]	0.226 [−6.00, 1.00]	0.0603 [−1.80, 1.36]
Missing	1 (0.0%)	586 (13.6%)	334 (2.5%)	172 (3.5%)
Externalising behaviours (*Z*)^a^	SDQ‐conduct and hyperactivity (5 years)	SDQ‐conduct and hyperactivity (5 years)	SDQ‐conduct and hyperactivity (5 years)	BPI‐Externalising problems (5 years)
Median [Min, Max]	0.214 [−4.27, 1.41]	0.126 [−4.45, 1.65]	0.239 [−4.41, 1.40]	0.164 [−1.75, 1.26]
Missing	1 (0.0%)	584 (13.5%)	335 (2.6%)	172 (3.5%)
Fluid IQ (*Z*)	Picture similarities (5 years)	Matrix Reasoning (7 years)	Picture similarities and pattern construction (5 years)	PIAT Mathematics (5 years)
Median [Min, Max]	−0.166 [−6.59, 2.75]	−0.247 [−2.59, 2.76]	0.161 [−4.62, 2.90]	0.0282 [−2.49, 2.40]
Missing	72 (0.8%)	288 (6.7%)	13 (0.1%)	653 (13.1%)
Crystal IQ (*Z*)	BAS Naming Vocab (5 years)	Peabody Picture Vocabulary Test (5 years)	BAS Naming Vocab (5 years)	Peabody Picture Vocabulary Test (5 years)
Median [Min, Max]	0.0616 [−4.69, 3.26]	0.156 [−5.11, 3.22]	0.0642 [−4.59, 3.09]	0.113 [−4.14, 3.34]
Missing	109 (1.3%)	176 (4.1%)	0 (0%)	344 (6.9%)
Child sex				
Male	4,406 (50.6%)	2,204 (51.1%)	6,671 (50.8%)	2,543 (51.2%)
Female	4,297 (49.4%)	2,112 (48.9%)	6,453 (49.2%)	2,425 (48.8%)
Familial income				
Low income	2,996 (34.4%)	1,257 (29.1%)	4,473 (34.1%)	963 (19.4%)
Medium income	3,370 (38.7%)	1,794 (41.6%)	4,682 (35.7%)	2,451 (49.3%)
High income	2,337 (26.9%)	1,265 (29.3%)	3,969 (30.2%)	1,554 (31.3%)
Mother's education level				
Non‐university educated	6,060 (69.6%)	2,544 (58.9%)	10,760 (82.0%)	2,895 (58.3%)
University educated	2,643 (30.4%)	1,772 (41.1%)	2,364 (18.0%)	2,073 (41.7%)

^a^ These measures were recoded from their original values so that a higher score would indicate a positive score/outcome (i.e. an easier infant temperament and improved behavioural outcomes).

### Ethical considerations

Cohorts had informed consent from parents and ethical reviews by the institutional review boards of Ohio State University and NORC at the University of Chicago, the South West MREC, Dublin's Committee of the Health Research Board and the Australian Health Ethics Committee (Christensen, Zubrick, Lawrence, Mitrou, & Taylor, 2014; Kelly, Sacker, Bono, Francesconi, & Marmot, 2011; Meeus et al., 2021; Shiely et al., 2017).

### Scale generation procedure

Predominantly, pre‐established scales developed for population‐based cohorts were used, see Table S1 for information on all scales and variables. When no pre‐established scale existed, scores were created based on individual items filled out by mothers (main analyses) or observer reports (sensitivity analyses). As this data were ordinal or binary, the R package ‘Gifi’ was used to conduct principal components analysis (Mair, De Leeuw, Groenen, & Mair, 2019). Items were included when factor loading scores were greater than 0.4. When there were multiple measures of a developmental outcome at the same age, principal component analyses were performed to create a total score (Vel Vignesh, Boolog, Bagyalakshmi, & Thilaga, 2024). Within each cohort, *Z* scores were created for all infant characteristics, parenting factors and developmental outcomes (*M* = 0, *SD* = 1).

### Individual infant characteristics

#### Temperament

Cohorts differed in measures of infant temperament but were measured at approximately 1 year old. We adopted the common definition of difficult infant temperament as being arrhythmic, withdrawing, low in adaptability, intense and emotionally negative (Carey & McDevitt, 1978). In MCS, the Carey Infant Temperament Scale was utilised. Mothers' answers on 14 items were summed, with a higher score indicating an easier temperament (Carey & McDevitt, 1978).

The LSAC utilised the Difficult Temperament Scale with three dimensions of cooperation, irritability and approach (Bergmeier, Skouteris, Horwood, Hooley, & Richardson, 2014). Mothers' answers were summed after the reverse coding of irritability subscale, with a higher total score indicating an easier temperament.

The NLSY79 measured temperament using a scale largely based on the Rothbart's Infant Behaviour Questionnaire (Rothbart, 1981). The ‘Difficulty’ composite score consists of 14 items answered by the mother which was then reversed coded (Padilla, Hines, & Ryan, 2020).

The GUI measured infant temperament using the ‘fussy‐difficult’ subscale of the Infant Characteristics Questionnaire Six‐Month Version. The subscale is a 6‐item subscale with mothers rating their infants' fussiness (Bates, 1980), which was then reversed coded.

#### Birthweight

In all cohorts, main carers provided data on birthweight at each cohort's first assessment point.

### Parenting factors

Items used in the main analyses were maternally reported at approximately 3 years of age. An overview of parenting scales, including observer‐reported items which were available in 3/4 cohorts and used in supplementary analyses, is shown in Table S1.

The definition of stimulating parenting as ‘having educational materials and engaging in enrichment activities thought to promote cognitive development and learning within the home’ was used (Simpkins et al., 2009). Furthermore, the definition of sensitive parenting as ‘being able to respond in a predictable, coherent, warm and accepting manner to a child's signals during daily interactions’ was used (Tarabulsy, 2016).

#### Stimulating parenting

In MCS, the principal component of six items that assessed stimulating parenting on a Likert scale was used, originally from the HOME short form (Bradley & Caldwell, 1984). In LSAC, 12 items from subscales of indoor and outdoor activities on a Likert scale were used (Hayes, 2015). The sub‐scores were averaged and then combined to create a total score. In NLSY79, maternal‐reported items from the HOME – Cognitive Stimulation subscale were used, consisting of up to 15 binary items (Mott, 2004). The total score was the mean score on all available items. In GUI, the principal component of seven items answered on a Likert scale was used.

#### Sensitive parenting

In MCS, the principal component of 22 items from the Straus Conflicts scale and the Pianta Child–Parent relationship scale (Pianta, 1992; Straus & Hamby, 1997) was used as the total score.

In LSAC, a parenting style measure that measured warmth (the mean of six items originally from the Child Rearing Questionnaire) (Paterson & Sanson, 1999) and hostility (the mean of five items originally from Early Childhood Longitudinal Study of Children – Birth Cohort) (Bethel, Green, Nord, Kalton, & West, 2005) was used. After reverse coding hostility, sensitive parenting was the combined score from both subscales (Zubrick, Lucas, Westrupp, & Nicholson, 2014).

In NLSY79, maternal‐reported items from HOME – Emotional Support subscale were used (Mott, 2004). Five items were summed together with any non‐binary items dichotomised.

In GUI, the same parenting style measures as in LSAC were used with an extra subscale of consistency (five items from the National Longitudinal Survey of Children and Youth) (Government of Canada, 2009). After reverse coding hostility, sensitive parenting was the averaged combined scores from the three subscales (Zubrick et al., 2014).

### Developmental outcomes

Cognitive performance was subdivided into fluid and crystallised IQ while behavioural outcomes were subdivided into internalising and externalising behaviours (Table S1). Outcomes were measured once, using standardised tests at approximately 5 years of age except for LSAC's fluid intelligence measure at 7 years.

#### Cognitive outcomes

Fluid intelligence was measured in the MCS using the principal component of scores on the British Ability Scales Picture Similarities and Pattern Construction tests (Elliott, 1996). In LSAC, WISC‐IV's Matrix Reasoning test was administered (O'Donnell, 2009). In NLSY79, the Peabody Individual Achievement Test‐ Mathematics subtest was used (Dunn & Markwardt, 1970). In GUI, the British Ability Scales' Picture Similarities test was used (Elliott, 1996).

Crystallised intelligence was measured in MCS and GUI using the British Ability Scales' Naming Vocabulary test (Elliott, 1996). In LSAC, Peabody Picture Vocabulary Test‐ third edition was used (Dunn & Dunn, 1997) while  in the NLSY79 the Peabody Picture Vocabulary Test‐ revised version was used (Dunn & Dunn, 1981).

#### Behavioural outcomes

In MCS, LSAC and GUI, the parent version of the Strengths and Difficulties Questionnaire was used, as reported by the mothers (Goodman, 1997). Combining the conduct and hyperactivity subscales from the questionnaire produces an externalising score while combining emotional problems and peer problems produces an internalising score. In the NLSY79, mothers completed the Behaviour Problems Index, where the internalising and externalising subscales were used (Peterson & Zill, 1986). For improved comparability with cognitive outcomes, scores were coded such that higher scores reflect fewer internalising or externalising problems.

#### Covariates

Three harmonizable covariates of child sex (Male = 1, Female = 0), the mother's education level (University educated = 1, Otherwise = 0) and the household income (*Z* standardised in each cohort) were used, with 4.1% missing covariate data imputed using a single imputation in MICE, with polytomous regression and predictive mean matching used for categorical and continuous variables respectively (van Buuren & Groothuis‐Oudshoorn, 2011).

### Statistical analyses

Initial analyses involved 16 multiple regressions (2 individual characteristics × 2 parenting factors × 4 developmental outcomes) in each cohort, resulting in 64 regressions using complete case analyses. To provide cohort‐pooled results, two types of data syntheses were then performed. First, two‐stage IPD syntheses were performed (IPD:2S) where the beta coefficients from each cohort's respective regression were combined using a random effects meta‐analysis methodology. By calculating the combined effects and *I* ^2^ values, consistent associations between developmental outcomes and individual characteristics, parenting factors and the respective interactions could be determined. From the IPD:2S syntheses, *p*‐values were adjusted for multiple comparisons using the false discovery rate correction (Yekutieli & Benjamini, 1999). In reporting the effect sizes of the combined beta coefficients, standardised betas between 0.10 and 0.29, 0.30 and 0.49 and 0.50 or greater were considered small, medium and large, respectively (Cohen, 2013). Subsequently, cohort data was pooled and 16 linear mixed models with random slopes were performed, akin to a one‐stage individual participant data (IPD:1S) meta‐analysis.

The main analysis to determine whether an analysis indicated additive effects, diathesis stress, vantage sensitivity or differential susceptibility was done via the competitive‐confirmatory approach, as outlined by Belsky et al. (2013) and Jolicoeur‐Martineau et al. (2020) using the LEGIT package in R (Jolicoeur‐Martineau et al., 2020). Instead of determining if an interaction term is significant, this methodology provides support for a specific model (e.g. diathesis stress) based on systematically fixing or freely estimating specific parameters in a multiple regression equation. From multiple competing model specifications, the model with the lowest AIC value is then thought to be optimal. For further details see Appendix S1. Five robustness tests were then performed on the IPD:1S. These were: (1) Roisman et al. (2012)'s approach of testing the interaction term for significance and then calculating the Proportion of the Interaction (POI) and Proportion affected (PA) metrics to determine the interaction pattern (see Appendix S2); (2) Testing for nonlinear associations between the infant characteristics, the parenting factors and the developmental outcomes instead of linear associations (see Appendix S3); (3) Testing the models without covariates; (4) Replacing the maternal‐reported parenting factors for observer reports; and (5) Modifying sensitive parenting by not including items measuring hostility.

## Results

### Demographics and attrition

All cohorts showed an even sex ratio of between 50.6% and 51.2% male (Table 1). The starting number of eligible infants in each cohort, according to our criteria of having valid birthweight or temperament data at wave 1, were 18,739 (MCS) (Connelly & Platt, 2014), 5,093 (LSAC) (Bayer et al., 2011), 5,936 (NLSY79) (Chen, 2012; Wright & Jackson, 2022) and 11,128 (GUI) (Castro, Kearney, & Layte, 2015), Table 1 provides the demographics for all children who were included in at least one analysis which shows that 70% (MCS), 85% (LSAC), 84% (NLSY79) and 78% (GUI) had been followed longitudinally with data on at least one infant characteristic, one parenting factor and one developmental outcome at approximate ages of 1, 3 and 5, respectively. Due to differing amounts of missing data on infant characteristics, parenting factors and developmental outcomes; the complete case analyses at the cohort level ranged from the lowest sample size of 3,353 to the maximum of 13,115. For the complete case analyses using pooled data, the minimum number of participants was 25,443 to a maximum of 30,360, with an average of 27,770 participants. For further information on the non‐standardised scores and correlation matrices between scores, see Tables S2 and S3.

### One‐stage individual participant data syntheses (IPD‐1S) results

**Figure 2:**
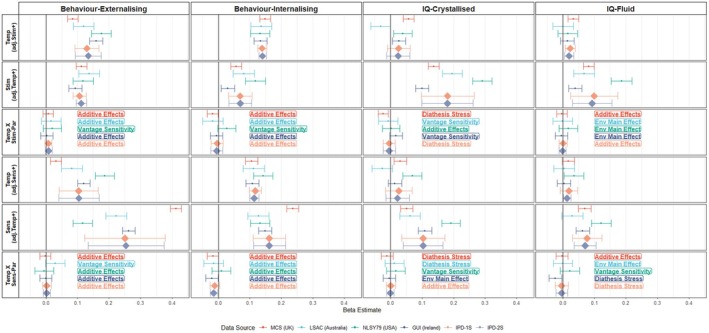
Infant temperament's association with developmental outcomes depending on stimulating or sensitive parenting. Figure shows the main effect of the individual characteristic (Infant temperament) – rows 1 and 4, the main effect of the environmental factors (Stim – stimulating parenting row 2, Sens – sensitive parenting row 5), and the respective interaction terms (rows 3 and 6) from either multiple regressions (cohort analyses), the linear mixed models (IPD‐1S) or the random effects syntheses (IPD‐2S). The nature of the interaction is decided upon using the competitive‐confirmatory approach, all performed as multiple regressions and is displayed as: additive effects (including both individual characteristic and environmental factor improves the AIC value), character main effect (only including the individual characteristic improves AIC value), env main effect (only including the environmental factor improves AIC) or the interaction term improves the AIC value and supports either diathesis stress, vantage sensitivity or differential susceptibility. The colour indicates the data source/cohort, MCS: Millennium Cohort Study – United Kingdom data (red), LSAC: Longitudinal Study of Australian Children – Australian data (light blue), NLSY79: National Longitudinal Survey of Youth 1979 child – United States of America data (green), GUI: Growing Up in Ireland‐ Republic of Ireland data (dark blue). IPD – Individual participant data pooled together in 1 stage (orange) or 2 stages (grey). All coefficients are adjusted for child sex, maternal education and family income. Error bars indicate 95% confidence intervals

**Figure 3:**
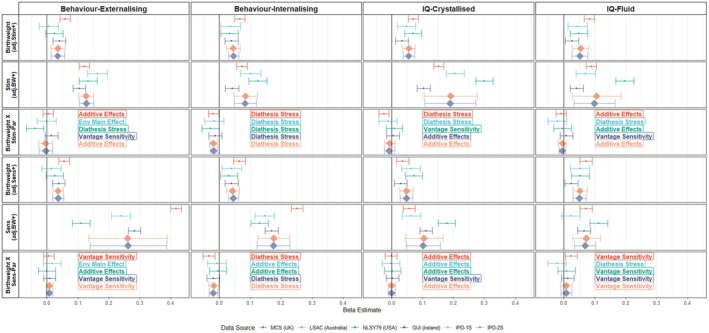
Birthweight's association with developmental outcomes depending on stimulating or sensitive parenting. Figure shows the beta coefficients of birthweight – rows 1 and 4, the main effect of the environmental factors (Stim – stimulating parenting row 2, Sens – sensitive parenting row 5) and the respective interaction terms (rows 3 and 6). The nature of the interaction, is decided upon using the competitive‐confirmatory approach and is displayed as: additive effects (including both individual characteristics and environmental factor improves the AIC value), character main effect (only including the individual characteristic improves AIC value), env main effect (only including the environmental factor improves AIC) or the interaction term improves the AIC value and supports either diathesis stress, vantage sensitivity or differential susceptibility. The colour indicates the data source/cohort, MCS: Millennium Cohort Study – United Kingdom data (red), LSAC: Longitudinal Study of Australian Children – Australian data (light blue), NLSY79: National Longitudinal Survey of Youth 1979 child – United States of America data (green), GUI: Growing Up in Ireland – Republic of Ireland data (dark blue). IPD – Individual participant data pooled together in 1 stage (orange) or 2 stages (grey). All coefficients are adjusted for child sex, maternal education and family income. Error bars indicate 95% confidence intervals

Using the IPD‐1S approach, cohort data were pooled and 16 linear mixed models and analyses using the competitive‐confirmatory approach were performed (Table 2, Figures 2 and 3). All infant characteristics, environmental factors and developmental outcomes were coded so that higher scores may be thought of as positive (e.g. an easier temperament, higher cognitive scores or fewer behavioural problems), allowing for evidence of additive effects, diathesis stress, vantage sensitivity or differential susceptibility to be more simply compared across analyses.

**Table 2:** Summary of IPD:1S results using differing methodologies

Robustness tests								
Developmental outcome	Infant characteristic	Parenting factor	Supported model main analysis^a^	Roisman et al. (2012)'s POI/PA methodology	Nonlinear testing	Without covariates	Observer reported parenting	Modified sensitive parenting
IQ‐Crystallised	Temperament	Stimulating	Diathesis stress	✗‐Env Main Effect	✗‐Additive effects	✔	✗‐Additive effects	✔
IQ‐Crystallised	Temperament	Sensitive	Additive effects	✗‐Env Main Effect	✔	✔	✗‐Diathesis stress	✔
IQ‐Crystallised	Birthweight	Stimulating	Additive effects	✔	✗‐Diathesis stress	✔	✔	✔
IQ‐Crystallised	Birthweight	Sensitive	Additive effects	✔	✔	✔	✔	✔
IQ‐Fluid	Temperament	Stimulating	Additive effects	✔	✔	✔	✗‐Diathesis stress	✔
IQ‐Fluid	Temperament	Sensitive	Diathesis stress	✗‐Env Main Effect	✗‐Additive effects	✔	✔	✔
IQ‐Fluid	Birthweight	Stimulating	Additive effects	✔	✔	✔	✔	✔
IQ‐Fluid	Birthweight	Sensitive	Vantage sensitivity	✗‐Additive Effects	✔	✔	✗‐Additive effects	✔
Behaviour‐Internalising	Temperament	Stimulating	Additive effects	✔	✔	✔	✔	✔
Behaviour‐Internalising	Temperament	Sensitive	Additive effects	✔	✔	✔	✔	✔
Behaviour‐Internalising	Birthweight	Stimulating	Diathesis stress	✔	✔	✔	✗‐Additive effects	✔
Behaviour‐Internalising	Birthweight	Sensitive	Diathesis stress	✗‐Additive effects	✔	✔	✗‐Additive effects	✔
Behaviour‐Externalising	Temperament	Stimulating	Additive effects	✔	✗‐Vantage sensitivity	✔	✔	✔
Behaviour‐Externalising	Temperament	Sensitive	Additive effects	✔	✗‐Vantage sensitivity	✔	✔	✔
Behaviour‐Externalising	Birthweight	Stimulating	Additive effects	✔	✔	✔	✔	✔
Behaviour‐Externalising	Birthweight	Sensitive	Vantage sensitivity	✗‐Additive effects	✗‐Additive effects	✔	✔	✔

^a^ The main analysis uses maternal reported parenting factors, including covariates of child sex, familial income and maternal education and when determining the optimal model uses linear regression via the LEGIT R package.

Beta coefficients for analyses considering temperament and birthweight in each cohort separately and after pooling the data are shown in Figures 2 and 3 respectively. For the IPD‐1S analyses, the competitive‐confirmatory approach indicated that the optimal models included interactions between infant characteristics and parenting factors in 6/16 analyses. Of these six, four supported diathesis stress with two analyses instead supporting vantage sensitivity. For the 10 other analyses, the additive effects model was optimal.

The robustness of the IPD‐1S main analyses was then examined by attempting to reproduce the findings with alternative methods (Table 2). The first check, determining whether the interaction term from a linear mixed model was significant and then calculating the POI and PA metrics, replicated the same model in 10/16 analyses. The simple slopes and Johnson‐Neyman plots from these models are shown in Figures S1 and S2, indicating that the infant characteristics of higher birthweight and easier temperament were positively associated with developmental outcomes, regardless of the level of the parenting factor. The second check involved testing for nonlinear associations between infant characteristics, parenting factors and developmental outcomes. It also found the same optimal model in 10/16 analyses. The third robustness check involved running the analyses without controlling for covariates. This, however, had no influence on which optimal model was found (16/16 reproduced findings), see Figures S1 and S2. The fourth check involved using observer‐reported (i.e. external interviewer‐reported) parenting rather than the originally used maternal reports. Despite only including 3/4 cohorts, the same optimal model was found in 10/16 analyses. The fifth check, modifying sensitive parenting by removing items measuring hostility, did not result in any changes. See Appendices S1–S3 for further information on the alternative methodologies. In sum, the IPD:1S main analyses finding additive effects were completely robust (i.e., across all robustness checks) in 5/10 analyses while diathesis‐stress and vantage‐sensitivity effects were never fully robust (0/4 and 0/2, respectively).

### Consistency of results across cohorts

In each cohort specifically, 16 multiple regressions and analyses using the competitive‐confirmatory approach were performed, resulting in a total of 64 cohort‐specific analyses. The competitive‐confirmatory approach indicated an optimal model with no interaction between the infant characteristic and the parenting factor in 33/64 analyses (Figures 2 and 3). 26/33 were optimal with inclusion of the infant characteristic and parenting factors (additive effects) while seven were optimal when only including the parenting factor (environment main effect). The 31 other analyses supported diathesis stress and vantage sensitivity 15 and 16 times, respectively. Cohort analyses were repeated without covariates, finding minimally more evidence for differential susceptibility (1/64 analyses) and additive effects (29/64 analyses) with less evidence for environment main effect models (4/64 analyses) (Figures S3 and S4).

When differentiating by infant characteristics, temperament less frequently interacted with parenting factors than birthweight (Figures 2 and 3). For temperament, vantage sensitivity and diathesis stress were supported seven and four times, respectively. For birthweight, the optimal model was vantage sensitivity and diathesis stress in 9/32 and 11/32 analyses, respectively. Table S4 demonstrates how often the same optimal model was found across cohorts; birthweight and simulating parenting predicting internalising behaviours was the single case where all cohorts indicated the same diathesis‐stress effect.

### Two‐stage IPD syntheses (IPD‐2S) results

To further evaluate pooled effects, 16 IPD‐2S syntheses were performed where the beta coefficients from each cohort's respective regression were combined (Table 3). Birthweight was significantly associated with all four outcomes whereas temperament was consistently significantly associated with behavioural outcomes (internalising and externalising behaviour problems) but inconsistently with fluid and crystallised intelligence. However, effect sizes for associations between infant characteristics and developmental outcomes were small. The largest beta indicated an easier temperament was associated with fewer internalising problems at 0.14 [95% CI = 0.13–0.15, *p* < .001].

**Table 3:** Results from the IPD:2S syntheses

Individual characteristic × parenting factor	Outcome	Beta individual characteristic	*I* ^2^ individual characteristic	Beta parent factor	*I* ^2^ parent factor	Beta interaction term	*I* ^2^ interaction term
Temperament × stimulating	IQ‐crystallised	0.02 [−0.01 to 0.06]	89.64	0.18 [0.10 to 0.26]	97.88	−0.00 [−0.02 to 0.02]	66.04
Temperament × sensitive	IQ‐crystallised	0.02 [−0.01 to 0.06]	88.69	0.10 [0.04 to 0.17]	96.02	−0.00 [−0.01 to 0.01]	4.95
Birthweight × stimulating	IQ‐crystallised	0.06 [0.04 to 0.07]	62.17	0.19 [0.11 to 0.27]	98.09	−0.01 [−0.02 to 0.01]	54.81
Birthweight × sensitive	IQ‐crystallised	0.05 [0.03 to 0.07]	64.89	0.10 [0.05 to 0.16]	95.25	0.00 [−0.01 to 0.01]	0.00
Temperament × stimulating	IQ‐fluid	0.02 [0.00 to 0.03]	32.70	0.09 [0.03 to 0.16]	96.07	−0.00 [−0.01 to 0.01]	0.00
Temperament × sensitive	IQ‐fluid	0.01 [0.00 to 0.03]	2.66	0.07 [0.04 to 0.11]	86.60	−0.00 [−0.02 to 0.01]	55.09
Birthweight × stimulating	IQ‐fluid	0.05 [0.03 to 0.08]	78.57	0.10 [0.03 to 0.16]	96.77	−0.00 [−0.02 to 0.01]	0.15
Birthweight × sensitive	IQ‐fluid	0.05 [0.03 to 0.07]	66.80	0.07 [0.03 to 0.10]	87.23	0.01 [−0.01 to 0.03]	54.97
Temperament × stimulating	Behaviour‐internalising	0.14 [0.13 to 0.15]	0.00	0.07 [0.03 to 0.11]	88.33	−0.00 [−0.02 to 0.01]	61.02
Temperament × sensitive	Behaviour‐internalising	0.11 [0.10 to 0.13]	25.89	0.16 [0.11 to 0.21]	93.84	−0.01 [−0.03 to 0.00]	0.85
Birthweight × stimulating	Behaviour‐internalising	0.05 [0.03 to 0.06]	53.24	0.08 [0.05 to 0.12]	88.48	−0.02 [−0.03 to 0.01]	0.00
Birthweight × sensitive	Behaviour‐internalising	0.05 [0.03 to 0.06]	45.50	0.18 [0.12 to 0.23]	94.87	−0.02 [−0.03 to 0.01]	26.22
Temperament × stimulating	Behaviour‐externalising	0.13 [0.09 to 0.18]	91.49	0.11 [0.10 to 0.13]	46.01	0.01 [−0.00 to 0.02]	0.00
Temperament × sensitive	Behaviour‐externalising	0.10 [0.04 to 0.17]	96.52	0.25 [0.13 to 0.37]	99.01	0.00 [−0.01 to 0.01]	2.88
Birthweight × stimulating	Behaviour‐externalising	0.04 [0.01 to 0.06]	71.74	0.13 [0.11 to 0.15]	72.89	−0.00 [−0.02 to 0.02]	71.46
Birthweight × sensitive	Behaviour‐externalising	0.04 [0.02 to 0.05]	56.72	0.26 [0.14 to 0.39]	99.14	0.01 [−0.00 to 0.02]	0.00

Regarding environmental factors, stimulating and sensitive parenting were consistently significantly associated with developmental outcomes but effect sizes were small. The largest beta indicated more sensitive parenting was associated with fewer externalising behaviours at 0.26 [95% CI = 0.14–0.39, *p* < .001]. Finally, there were three significant interaction terms, all with the outcome of internalising behaviours. These were birthweight interacting with stimulating parenting (beta = −0.02 [95% CI −0.03 to 0.01], *p* = .003) and sensitive parenting (beta = −0.02 [95% CI −0.03 to 0.01], *p* = .009), and temperament interacting with sensitive parenting (beta = −0.01 [95% CI −0.03 to 0.00], *p* = .025) when predicting internalising problems.

## Discussion

We investigated whether infant characteristics and parenting factors are associated with cognition and behaviour in a way that supports a specific theoretical model of either additive effects, diathesis stress, vantage sensitivity or differential susceptibility. Overall, the most consistent evidence was found for additive effects, indicating that infant characteristics and parenting factors are consistently but largely independently associated with developmental outcomes. In a minority of analyses, interactions indicating diathesis stress and vantage sensitivity emerged. The diathesis‐stress pattern appeared largely domain‐specific to internalising behaviours and consistent across cohorts. While three significant interactions were found when using the IPD‐2S approach, their effect sizes were small relative to their respective additive effects. Finally, differential susceptibility, which challenges traditional concepts of risk and resilience (Ellis, Boyce, Belsky, Bakermans‐Kranenburg, & van Ijzendoorn, 2011), was rarely supported.

Infants with difficult temperament were expected to show poorer outcomes in negative environments but show particularly good outcomes in positive environments, relative to those with easier temperament. However, analyses including covariates provided no empirical support for temperament being a marker of differential susceptibility. Instead, infants with easy temperaments benefitted more often from positive environments, indicating vantage sensitivity. When compared to past temperament studies investigating similar environmental factors and developmental outcomes (Blair, 2002), including a past meta‐regression (Slagt et al., 2016), our non‐converging results may result from multiple methodological decisions. For example, our main analysis utilised a competitive‐confirmatory approach of model comparisons to determine which theoretical model was best supported. Instead, prior studies using meta‐regressions compare the relative associations between positive environments and positive outcomes (both on a scale from debatably neutral to positive) versus negative environments and negative outcomes (both on a scale from debatably neutral to negative) for infants with different temperaments (Slagt et al., 2016). This is potentially critical, as scales of behaviour problems range from the negative pole of many problems to a debatably neutral pole of no problems, without considering positive well‐being. Our focusing on a restricted range of behavioural outcomes potentially reduced the chances of finding differential susceptibility interaction patterns (Pluess, Stevens, & Belsky, 2013). While combining mental well‐being and mental illness into one dimension may increase the chances of finding certain interaction patterns, for most of the constructs within the realm of mental health there appears consensus that they ‘constitute separate but correlated unipolar dimensions’, and thus should remain separated (Keyes, 2005).

Our results surrounding temperament largely agree with prior studies where there was no evidence for differential susceptibility regarding parent‐reported psychopathology or cognitive performance (Roisman et al., 2012). Roisman et al. (2012) found evidence for differential susceptibility for teacher‐reported outcomes but the younger age of the participants in the current analyses meant this could not be tested. Overall, results suggest that for infant temperament, additive effects of the environment and to a lesser extent, vantage sensitivity and diathesis stress, are the models most commonly found for outcome at 5 years.

Regarding the other infant characteristics of interest, results were consistent with previous literature suggesting that lower birthweight is associated with lower cognitive performance and more behavioural problems (Gu et al., 2017; Jaekel et al., 2015). While non‐interactive effects were more common, 11/32 cohort‐specific analyses indicated that birthweight is a vulnerability factor that interacts with parenting factors, indicative of diathesis stress. Furthermore, the IPD‐1S analyses indicated evidence of diathesis stress for birthweight and both parenting factors on internalising behaviours. This was further supported by IPD‐2S analyses indicating significant negative interaction terms with little cohort heterogeneity. These results are, therefore, concordant with findings of extremely low birthweight adults being more negatively affected by environmental risks than normal birthweight controls for internalising outcomes (Van Lieshout et al., 2018). Thus, lower birthweight appears to be a marker of vulnerability for internalising behaviours, but potentially nullified if parenting is sufficient in quality.

Strengths of the current study include the utilisation of IPD synthesis to test the domain generality and universality of interaction effects regarding individual characteristics, environmental factors and developmental outcomes. In addition, this analysis was preregistered and used open‐access data, thought to increase transparency and reproducibility (John, Loewenstein, & Prelec, 2012). Additional non‐preregistered analyses were conducted as robustness checks, indicating considerable overlap between the main analyses and these subsequent post‐hoc analyses. This largely supports past findings suggesting a lack of difference between analyses using maternal or observer‐reported parenting factors (Slagt et al., 2016). Overall, the systematic testing of factors across large cohorts provides a novel and comprehensive analysis of how and to what extent birthweight and infant temperament are associated with later development.

This study also has limitations. First, cohort differences potentially reduced the ability to make direct comparisons. For example, some cohorts considered a larger scope of parenting behaviours than others, resulting in the distribution of scales sometimes differing in shape and spread, potentially inaccurately measuring extreme levels of parenting. Second, future research may consider genetic markers rather than behavioural markers such as infant temperament (Keers et al., 2016), especially as temperament may only have moderate long‐term stability (Bornstein et al., 2015). To harmonise the data across cohorts, to avoid questions of reverse causality (Rutter, 2013) and to follow similar past research (Roisman et al., 2012), the individual characteristics, the maternal‐reported parenting factors and the developmental outcomes were measured at ages 1, 3 and 5, respectively. It is possible that the age of assessment of the variables influences which interaction patterns are found (Rioux et al., 2016). In addition, developmental outcomes are also influenced by father behaviour (Hennigar & Cabrera, 2024), which should be integrated into future studies. Similarly, the utilisation of causal inference methodologies may be beneficial (Biazoli Jr., Sato, & Pluess, 2024; Igelström et al., 2022).

To conclude, this systematic test of the interplay between infant characteristics and parenting factors found more evidence for relatively small, additive associations of birthweight, temperament and parenting with developmental outcomes rather than supporting a specific interaction effect. While limited to internalising behaviours, a consistent cross‐cohort interaction effect suggests that in higher quality environments, children with lower birthweight can reach similar levels to those born at higher birthweights but not surpass them (diathesis stress). This finding needs buttressing with intervention studies to determine whether increasing stimulating and sensitive parenting via intervention results in improved developmental effects for the whole population of children but in particular for those with lower birthweight.

## Ethical considerations

Cohorts had informed consent from parents and ethical reviews by the institutional review boards of Ohio State University and NORC at the University of Chicago, the South West MREC, Dublin's Committee of the Health Research Board and the Australian Health Ethics Committee (Christensen et al., 2014; Kelly et al., 2011; Meeus et al., 2021; Shiely et al., 2017).

**Key points**

- What's known: Many past studies reported interactions between infant characteristics and parenting when predicting developmental outcomes, indicative of the theoretical models of diathesis stress, differential susceptibility or vantage sensitivity.
- What's new: We found little evidence for robust interactive effects across countries, developmental domains and analytic choices when using individual participant data from numerous population‐representative cohorts.
- What's relevant: Parenting's associations with later child outcomes are largely not moderated by infant characteristics, indicating parenting interventions may help all children similarly.

## Supporting information


**Table S1.** Extra scales and item information.
**Appendix S1.** Further description of the competitive‐confirmatory approach.
**Appendix S2.** Further description of the proportion affected (PA) and proportion of the interaction (POI) metrics by Roisman et al. (2012).
**Table S2.** Extended demographics using raw variables from cohorts.
**Table S3.** Correlation matrix of all main variables.
**Table S4.** Optimal model consistency across cohorts (4 = same optimal model in all 4 cohorts).
**Appendix S3.** Nonlinear testing.
**Figure S1.** Johnson–Neyman plots from the 16 IPD:1S analyses.
**Figure S2.** Simple slope plots from the 16 IPD:1S analyses.
**Figure S3.** Infant temperament's association with developmental outcomes depending on stimulating or sensitive parenting‐without covariates.
**Figure S4.** Birthweight's association with developmental outcomes depending on stimulating or sensitive parenting‐ without covariates.

## Data Availability

The data used in this publication is open access but must be requested from each cohort's respective websites: MCS: https://cls.ucl.ac.uk/cls‐studies/millennium‐cohort‐study/. GUI: https://www.growingup.gov.ie/. LSAC: https://growingupinaustralia.gov.au/. NLSY79: https://www.nlsinfo.org/content/cohorts/nlsy79‐children. The code used for this analysis can be found here: https://osf.io/ynpuk.
