# Supplementary material for: Interactions between infant characteristics and parenting factors rarely replicate across cohorts and developmental domains
Source: J Child Psychol Psychiatry. 2025 Mar 10;66(8):1234–48. doi: 10.1111/jcpp.14149 (PMC12267674; doi:10.1111/jcpp.14149)
Supplement: Supplementary file 1 — Table S1. Extra scales and item information. Appendix S1. Further description of the competitive‐confirmatory approach. Appendix S2. Further description of the proportion affected (PA) and proportion of the interaction (POI) metrics by Roisman et al. (2012). Table S2. Extended demographics using raw variables from cohorts. Table S3. Correlation matrix of all main variables. Table S4. Optimal model consistency across cohorts (4 = same optimal model in all 4 cohorts). Appendix S3. Nonlinear testing. Figure S1. Johnson–Neyman plots from the 16 IPD:1S analyses. Figure S2. Simple slope plots from the 16 IPD:1S analyses. Figure S3. Infant temperament's association with developmental outcomes depending on stimulating or sensitive parenting‐without covariates. Figure S4. Birthweight's association with developmental outcomes depending on stimulating or sensitive parenting‐ without covariates. [file JCPP-66-1234-s001.docx]

Contents

[**Table S1-Extra scales and item information** 2](#_Toc179998619)

[**Appendix S1- Further Description of the Competitive Confirmatory Approach** 34](#_Toc179998620)

[**Appendix S2- Further Description of the Proportion Affected (PA) and Proportion of the Interaction(POI) Metrics by Roisman (2012)** 34](#_Toc179998621)

[**Table S2 Extended Demographics using raw variables from cohorts** 35](#_Toc179998622)

[**Table S3 Correlation matrix of all main variables** 37](#_Toc179998623)

[**Table S4 Optimal model consistency across cohorts (4= same optimal model in all 4 cohorts)** 39](#_Toc179998624)

[**Appendix S3 Nonlinear Testing** 42](#_Toc179998625)

[Model 1 Plot 47](#_Toc179998626)

[Model 2 Plot 48](#_Toc179998627)

[Model 3 Plot 48](#_Toc179998628)

[Model 4 Plot 49](#_Toc179998629)

[Model 5 Plot 50](#_Toc179998630)

[Model 6 Plot 51](#_Toc179998631)

[Model 7 Plot 52](#_Toc179998632)

[Model 8 Plot 53](#_Toc179998633)

[Model 9 Plot 54](#_Toc179998634)

[Model 10 Plot 55](#_Toc179998635)

[Model 11 Plot 56](#_Toc179998636)

[Model 12 Plot 57](#_Toc179998637)

[Model 13 Plot 58](#_Toc179998638)

[Model 14 Plot 59](#_Toc179998639)

[Model 15 Plot 60](#_Toc179998640)

[Model 16 Plot 61](#_Toc179998641)

[**Figure S1: Johnson-Neyman plots from the 16 IPD:1S analyses** 63](#_Toc179998642)

[**Figure S2: Simple slope plots from the 16 IPD:1S analyses** 64](#_Toc179998643)

[**Figure S3: Infant temperament’s association on developmental outcomes depending on stimulating or sensitive parenting- without covariates** 65](#_Toc179998644)

[**Figure S4: Birthweight’s association on developmental outcomes depending on stimulating or sensitive parenting- without covariates** 66](#_Toc179998645)

Please see <https://osf.io/732nx> for the R markdown, interactive version of all supplementary material and the pre-registration.

# **Table S1-Extra scales and item information**

| Cohort | Measure | Item info | Item variable name | Parent or Observer Reported | Use in Analyses | Notes |
| --- | --- | --- | --- | --- | --- | --- |
| GUI | Crystal IQ | Total ability score for naming vocabulary | b3_nvabscore | Observer | Z scored | Test items consist of coloured pictures of objects shown one at a time and the cohort member was asked to name. The interviewer showed the cohort member a picture in the BAS easel and asked “What is this?” The cohort member responds verbally, recorded by the interviewer |
| LSAC | Crystal IQ | PPVT score | cppvt | Observer | Z scored | A book with 40 plates of display pictures was used. The child points to (or says the number of) a picture that best represents the meaning of the word read out by the interviewer |
| MCS | Crystal IQ | Naming Vocabulary ability score | CCNVABIL | Observer | Z scored | Test items consist of coloured pictures of objects shown one at a time and the cohort member was asked to name. The interviewer showed the cohort member a picture in the BAS easel and asked “What is this?” The cohort member responds verbally, recorded by the interviewer |
| NLSY79 | Crystal IQ | PPVT score | PPVTZ | Observer | Z scored | The assessment consists of 175 vocabulary items of generally increasing difficulty. The child nonverbally selects one of four pictures which best describes a particular word's meaning. |
| GUI | Externalising problems | SDQ Hyperactivity subscale | b3_sdqhyper | Parent | rowmeans and then Z scored | combined scores on the conduct and hyperactivity subscales (both 5 items) produce a total externalising score |
| GUI | Externalising problems | SDQ Conduct subscale | b3_sdqconduct | Parent | rowmeans and then Z scored | combined scores on the conduct and hyperactivity subscales (both 5 items) produce a total externalising score |
| LSAC | Externalising problems | SDQ Hyperactivity subscale | cahypr | Parent | rowmeans and then Z score | combined scores on the conduct and hyperactivity subscales (both 5 items) produce a total externalising score |
| LSAC | Externalising problems | SDQ Conduct subscale | caconda | Parent | rowmeans and then Z score | combined scores on the conduct and hyperactivity subscales (both 5 items) produce a total externalising score |
| MCS | Externalising problems | SDQ Conduct Problems | CCONDUCT | Parent | rowmeans and then Z score | combined scores on the conduct and hyperactivity subscales (both 5 items) produce a total externalising score |
| MCS | Externalising problems | SDQ Hyperactivity | CHYPER | Parent | rowmeans and then Z score | combined scores on the conduct and hyperactivity subscales (both 5 items) produce a total externalising score |
| NLSY79 | Externalising problems | BPI: Externalising score | BPEXTP | Parent | Z scored | 10 items measuring the frequency, range, and type of childhood behaviour problems |
| GUI | Fluid IQ | Total ability score for picture similarities | b3_psabscore | Observer | Z scored | Cohort member was shown a row of four pictures or designs, and the cohort member placed a fifth card below the stimulus picture it best matched |
| LSAC | Fluid IQ | Matrix reasoning score | dmatreas | Observer | Z scored | This test of non-verbal intelligence presents the child with an incomplete set of diagrams (an item) and requires them to select the picture that completes the set from five different options |
| MCS | Fluid IQ | Picture Similarities ability score | CCPSABIL | Observer | PCA and then Z scored | Picture Similarities: cohort member was shown a row of four pictures or designs, and the cohort member placed a fifth card below the stimulus picture it best matched |
| MCS | Fluid IQ | Pattern Construction ability score | CCPCABIL | Observer | PCA and then Z scored | Pattern construction tests: a pattern was presented to the cohort member, and the cohort member was asked to replicate the pattern using flat foam squares or solid plastic cubes with black and yellow patterns on each side |
| NLSY79 | Fluid IQ | PIAT Math Score | MATH | Observer | Z scored | PIAT Mathematics: 84 multiple-choice items of increasing difficulty. It begins with such early skills as recognizing numerals and progresses to measuring advanced concepts in geometry and trigonometry. The child looks at each problem on an easel page and then chooses an answer by pointing to or naming one of four answer options |
| GUI | Internalising problems | SDQ Peer problems subscale | b3_sdqpeerprobs | Parent | rowmeans and then Z scored | combined subscale of emotional problems and peer problems (both 5 items) produce a total internalising score |
| GUI | Internalising problems | SDQ Emotional subscale | b3_sdqemotional | Parent | rowmeans and then Z scored | combined subscale of emotional problems and peer problems (both 5 items) produce a total internalising score |
| LSAC | Internalising problems | SDQ Peer problems subscale | capeer | Parent | rowmeans and then Z score | combined subscale of emotional problems and peer problems (both 5 items) produce a total internalising score |
| LSAC | Internalising problems | SDQ Emotional subscale | caemot | Parent | rowmeans and then Z score | combined subscale of emotional problems and peer problems (both 5 items) produce a total internalising score |
| MCS | Internalising problems | SDQ Emotional Symptoms | CEMOTION | Parent | rowmeans and then Z score | combined subscale of emotional problems and peer problems (both 5 items) produce a total internalising score |
| MCS | Internalising problems | SDQ Peer Problems | CPEER | Parent | rowmeans and then Z score | combined subscale of emotional problems and peer problems (both 5 items) produce a total internalising score |
| NLSY79 | Internalising problems | BPI: internalising score | BPINTP | Parent | Z scored | 10 items measuring the frequency, range, and type of childhood behaviour problems |
| GUI | Child_Sex | Child's sex | MMa5ap2 | Parent | Covariate | NA |
| LSAC | Child_Sex | Child's sex | zf02m1 | Parent | Covariate | NA |
| MCS | Child_Sex | Child's sex | AHCSEX00 | Parent | Covariate | NA |
| NLSY79 | Child_Sex | Child's sex | CSEX_XRND | Parent | Covariate | NA |
| GUI | household_income | Equivalised Household Annual Income | Equivinc | Parent | Covariate | NA |
| LSAC | household_income | Combined yearly income before tax | afn05 | Parent | Covariate | NA |
| MCS | household_income | Predicted weekly net family income | AOEDEX00 | Parent | Covariate | NA |
| NLSY79 | household_income | Total Net Family Income | TNFI | Parent | Covariate | NA |
| GUI | Maternal_Education | Mother's education at 9 months | FF13 | Parent | Covariate | NA |
| LSAC | Maternal_Education | Mother's education at 1 year | afd08m3a | Parent | Covariate | NA |
| MCS | Maternal_Education | Highest academic qualification | APACQU00 | Parent | Covariate | NA |
| NLSY79 | Maternal_Education | Number of years education by mother | HGCDOI | Parent | Covariate | NA |
| GUI | Sensitive Parenting | Warmth subscale from PCG | bpc2_warmth | Parent | rowmeans and then Z scored | NA |
| GUI | Sensitive Parenting | Hostility subscale from PCG | bpc2_hostility | Parent | rowmeans and then Z scored | NA |
| GUI | Sensitive Parenting | Consistency subscale from PCG | bpc2_consistency | Parent | rowmeans and then Z scored | NA |
| GUI | Sensitive Parenting | Often express affection | NA | Parent | subscale scores used | NA |
| GUI | Sensitive Parenting | Often hug or hold child | NA | Parent | subscale scores used | NA |
| GUI | Sensitive Parenting | Often tell child how happy makes you? | NA | Parent | subscale scores used | NA |
| GUI | Sensitive Parenting | Often close times together with child? | NA | Parent | subscale scores used | NA |
| GUI | Sensitive Parenting | Often do you enjoy doing things with this child? | NA | Parent | subscale scores used | NA |
| GUI | Sensitive Parenting | Often feel close to child both when happy and upset? | NA | Parent | subscale scores used | NA |
| GUI | Sensitive Parenting | angry with child | NA | Parent | subscale scores used | NA |
| GUI | Sensitive Parenting | shouted at child | NA | Parent | subscale scores used | NA |
| GUI | Sensitive Parenting | When child cries, gets on nerves | NA | Parent | subscale scores used | NA |
| GUI | Sensitive Parenting | lost temper with child | NA | Parent | subscale scores used | NA |
| GUI | Sensitive Parenting | left child alone i when he/she was particularly upset | NA | Parent | subscale scores used | NA |
| GUI | Sensitive Parenting | Make sure completes requests | NA | Parent | subscale scores used | NA |
| GUI | Sensitive Parenting | Punish Study Child | NA | Parent | subscale scores used | NA |
| GUI | Sensitive Parenting | Study Child gets away unpunished | NA | Parent | subscale scores used | NA |
| GUI | Sensitive Parenting | Study Child gets out of punishment | NA | Parent | subscale scores used | NA |
| GUI | Sensitive Parenting | Study Child ignores punishment | NA | Parent | subscale scores used | NA |
| LSAC | Sensitive Parenting | Spontaneously praises Study Child | bpa06a | Observer | rowmeans and then Z scored | NA |
| LSAC | Sensitive Parenting | Scolds, etc. Study Child | bpa06b2 | Observer | rowmeans and then Z scored | NA |
| LSAC | Sensitive Parenting | Study Child was unkempt | bpa06c | Observer | rowmeans and then Z scored | NA |
| LSAC | Sensitive Parenting | Hug Study Child | bpa03m2 | Parent | Subscale Scores Combined | NA |
| LSAC | Sensitive Parenting | Express happiness to Study Child | bpa03m3 | Parent | Subscale Scores Combined | NA |
| LSAC | Sensitive Parenting | Warm encounters with Study Child | bpa03m4 | Parent | Subscale Scores Combined | NA |
| LSAC | Sensitive Parenting | Enjoy doing things with Study Child | bpa03m5 | Parent | Subscale Scores Combined | NA |
| LSAC | Sensitive Parenting | Close when happy or upset | bpa03m6 | Parent | Subscale Scores Combined | NA |
| LSAC | Sensitive Parenting | Display physical affection | bpa03m1 | Parent | Subscale Scores Combined | NA |
| LSAC | Sensitive Parenting | Warm Parenting | bmwarm | Parent | Warm and Hostile subscales are Z scored, combined, and then z scored again | NA |
| LSAC | Sensitive Parenting | Angry with Study Child | bpa04m1 | Parent | Subscale Scores Combined | NA |
| LSAC | Sensitive Parenting | Shout at Study Child | bpa04m2 | Parent | Subscale Scores Combined | NA |
| LSAC | Sensitive Parenting | Gets on nerves when crying | bpa04m3 | Parent | Subscale Scores Combined | NA |
| LSAC | Sensitive Parenting | Lose temper with Study Child | bpa04m4 | Parent | Subscale Scores Combined | NA |
| LSAC | Sensitive Parenting | Leave Study Child alone when upset | bpa04m5 | Parent | Subscale Scores Combined | NA |
| LSAC | Sensitive Parenting | Hostile Parenting | bmhost | Parent | Warm and Hostile subscales are Z scored, combined, and then z scored again | NA |
| MCS | Sensitive Parenting | Mother's voice positive when speaking to child | BCSPEA00 | Observer | rowmeans and then Z scored | NA |
| MCS | Sensitive Parenting | Mother converses at least twice with child | BCMCON00 | Observer | rowmeans and then Z scored | NA |
| MCS | Sensitive Parenting | Mother answers child's questions verbally | BCANSW00 | Observer | rowmeans and then Z scored | NA |
| MCS | Sensitive Parenting | Mother praises child spontaneously | BCPRAI00 | Observer | rowmeans and then Z scored | NA |
| MCS | Sensitive Parenting | Mother caresses or kisses child | BCKISS00 | Observer | rowmeans and then Z scored | NA |
| MCS | Sensitive Parenting | Mother introduces interviewer to child | BCINTI00 | Observer | rowmeans and then Z scored | NA |
| MCS | Sensitive Parenting | Mother scolded child more than once | BCSCOL00 | Observer | rowmeans and then Z scored | NA |
| MCS | Sensitive Parenting | Mother used physical restraint on child | BCPHYS00 | Observer | rowmeans and then Z scored | NA |
| MCS | Sensitive Parenting | Mother slapped or spanked child | BCSLAP00 | Observer | rowmeans and then Z scored | NA |
| MCS | Sensitive Parenting | Parent kept child in vision | BCSEEC00 | Observer | rowmeans and then Z scored | NA |
| MCS | Sensitive Parenting | Ignore child if being naughty | BPDIIG00 | Parent | Princals used to create total Z score | NA |
| MCS | Sensitive Parenting | How often smacks Study Child when naughty | BPDISM00 | Parent | Princals used to create total Z score | NA |
| MCS | Sensitive Parenting | How often shouts at Study Child when naughty | BPDISH00 | Parent | Princals used to create total Z score | NA |
| MCS | Sensitive Parenting | How often sends Study Child to bedroom/naughty chair | BPDIBN00 | Parent | Princals used to create total Z score | NA |
| MCS | Sensitive Parenting | Take away treats if child being naughty | BPDITR00 | Parent | Princals used to create total Z score | NA |
| MCS | Sensitive Parenting | How often tells Study Child off when naughty | BPDITE00 | Parent | Princals used to create total Z score | NA |
| MCS | Sensitive Parenting | How often bribes Study Child when naughty | BPDIBR00 | Parent | Princals used to create total Z score | NA |
| MCS | Sensitive Parenting | Warm, affectionate relationship with child | BPPIAW00 | Parent | Princals used to create total Z score | NA |
| MCS | Sensitive Parenting | Struggle with child | BPPIST00 | Parent | Princals used to create total Z score | NA |
| MCS | Sensitive Parenting | Child will seek comfort from me | BPPICO00 | Parent | Princals used to create total Z score | NA |
| MCS | Sensitive Parenting | Child uncomfortable with physical affection | BPPIPT00 | Parent | Princals used to create total Z score | NA |
| MCS | Sensitive Parenting | Child values relationship with me | BPPIVA00 | Parent | Princals used to create total Z score | NA |
| MCS | Sensitive Parenting | When praise child he/she beams with pride | BPPIBP00 | Parent | Princals used to create total Z score | NA |
| MCS | Sensitive Parenting | Child spontaneously shares information with me | BPPISI00 | Parent | Princals used to create total Z score | NA |
| MCS | Sensitive Parenting | Child easily becomes angry with me | BPPIAN00 | Parent | Princals used to create total Z score | NA |
| MCS | Sensitive Parenting | Easy to be in tune with child's feelings | BPPIET00 | Parent | Princals used to create total Z score | NA |
| MCS | Sensitive Parenting | Child angry or resistant after disciplined | BPPIAR00 | Parent | Princals used to create total Z score | NA |
| MCS | Sensitive Parenting | Dealing with child drains my energy | BPPIDE00 | Parent | Princals used to create total Z score | NA |
| MCS | Sensitive Parenting | Child bad mood, in for a long day | BPPIBD00 | Parent | Princals used to create total Z score | NA |
| MCS | Sensitive Parenting | Child's feeling can be unpredictable towards me | BPPIUC00 | Parent | Princals used to create total Z score | NA |
| MCS | Sensitive Parenting | Child is sneaky/manipulative with me | BPPISM00 | Parent | Princals used to create total Z score | NA |
| MCS | Sensitive Parenting | Child openly shares feelings/experiences | BPPISE00 | Parent | Princals used to create total Z score | NA |
| NLSY79 | Sensitive Parenting | Interviewer: Mother spontaneously spoke to child twice or more (excluding scolding)? | CSOB-2A | Observer | rowmeans and then Z scored | NA |
| NLSY79 | Sensitive Parenting | Interviewer: Mother responded verbally to child's speech? | CSOB-3A | Observer | rowmeans and then Z scored | NA |
| NLSY79 | Sensitive Parenting | Interviewer: Mother caressed, kissed, or hugged child at least once? | CSOB-4A | Observer | rowmeans and then Z scored | NA |
| NLSY79 | Sensitive Parenting | Interviewer: Mother slapped or spanked child at least once? | CSOB-5A | Observer | rowmeans and then Z scored | NA |
| NLSY79 | Sensitive Parenting | Interviewer: Mother interfered w/ child's actions or restricted child from exploring >= 3 times? | CSOB-6A | Observer | rowmeans and then Z scored | NA |
| NLSY79 | Sensitive Parenting | Interviewer: Mother introduced interviewer to child by name? | CSOB-5B | Observer | rowmeans and then Z scored | NA |
| NLSY79 | Sensitive Parenting | Interviewer: Mother's voice conveyed positive feeling about child? | CSOB-8B | Observer | rowmeans and then Z scored | NA |
| NLSY79 | Sensitive Parenting | Interviewer: Mother kept child in view/ could see child/ looked at him/her often? | CSOB-8A | Observer | rowmeans and then Z scored | NA |
| NLSY79 | Sensitive Parenting | How often does child eat a meal with both you and his/her father/step/father-figure? | HOME-A15 | Parent | rowmeans and then Z scored | NA |
| NLSY79 | Sensitive Parenting | How often do you talk to child while you are working? | HOME-A16 | Parent | rowmeans and then Z scored | NA |
| NLSY79 | Sensitive Parenting | About how many times, if any, have you had to spank child in the past week? | HOME-A18 | Parent | rowmeans and then Z scored | NA |
| NLSY79 | Sensitive Parenting | Number of Hours TV | MS-HOME-B07B | Parent | rowmeans and then Z scored | NA |
| NLSY79 | Sensitive Parenting | Choice with food | MS-HOME-B06 | Parent | rowmeans and then Z scored | NA |
| GUI | Stimulating Parenting | Read to study child - days per week | bpc2E3a | Parent | Princals used to create total Z score | NA |
| GUI | Stimulating Parenting | ABC or alphabet - days per week | bpc2E3b | Parent | Princals used to create total Z score | NA |
| GUI | Stimulating Parenting | Numbers or counting - days per week | bpc2E3c | Parent | Princals used to create total Z score | NA |
| GUI | Stimulating Parenting | Songs, poems or nursery rhymes - days per week | bpc2E3d | Parent | Princals used to create total Z score | NA |
| GUI | Stimulating Parenting | Play games [board games, jigsaws, card games etc] - days per week | bpc2E3e | Parent | Princals used to create total Z score | NA |
| GUI | Stimulating Parenting | Paint, draw, colour, or play with play-doh at home - days per week | bpc2E3f | Parent | Princals used to create total Z score | NA |
| GUI | Stimulating Parenting | How many children’s books in your home now, including any library books? | bpc2E4 | Parent | Not use due to low loading | NA |
| LSAC | Stimulating Parenting | Told the Study Child a story | 2/3 - P1 - F2F F1.1 - Told the SC a story | Parent | Subscale Scores Combined | NA |
| LSAC | Stimulating Parenting | Drawn picture with Study Child | 2/3 - P1 - F2F F1.2 - Drawn picture with SC | Parent | Subscale Scores Combined | NA |
| LSAC | Stimulating Parenting | Music with Study Child | 2/3 - P1 - F2F F1.3 - Music with SC | Parent | Subscale Scores Combined | NA |
| LSAC | Stimulating Parenting | Toys/games with Study Child | 2/3 - P1 - F2F F1.4 - Toys/games with SC | Parent | Subscale Scores Combined | NA |
| LSAC | Stimulating Parenting | Everyday activities with Study Child | 2/3 - P1 - F2F F1.5 - Everyday activities with SC | Parent | Subscale Scores Combined | NA |
| LSAC | Stimulating Parenting | Played outdoors with Study Child | 2/3 - P1 - F2F F1.6 - Played outdoors with SC | Parent | Subscale Scores Combined | NA |
| LSAC | Stimulating Parenting | Read to Study Child | 2/3 - P1 - F2F F1.7 - Read to SC | Parent | Subscale Scores Combined | NA |
| LSAC | Stimulating Parenting | Home Activities index | 2/3 - P1 - Home Activities index | Parent | Home activities and out of home activities are combined and then Z scored | NA |
| LSAC | Stimulating Parenting | Gone to a playground/pool | 2/3 - P1 - F2F F5.1 - Gone to a playground/pool | Parent | Subscale Scores Combined | NA |
| LSAC | Stimulating Parenting | Gone to concert, museum etc. | 2/3 - P1 - F2F F5.2 - Gone to concert, museum etc. | Parent | Subscale Scores Combined | NA |
| LSAC | Stimulating Parenting | Gone to movies/sporting event | 2/3 - P1 - F2F F5.3 - Gone to movies/sporting event | Parent | Subscale Scores Combined | NA |
| LSAC | Stimulating Parenting | Attended a religious service | 2/3 - P1 - F2F F5.4 - Attended a religious service | Parent | Subscale Scores Combined | NA |
| LSAC | Stimulating Parenting | Visited library | 2/3 - P1 - F2F F5.5 - Visited library | Parent | Subscale Scores Combined | NA |
| LSAC | Stimulating Parenting | Out of home activities index (v2) | 2/3 - Out of home activities index (v2) | Parent | Home activities and out of home activities are combined and then Z scored | NA |
| MCS | Stimulating Parenting | Child's in-home play environment safe | BCENVI00 | Observer | rowmeans and then Z scored | NA |
| MCS | Stimulating Parenting | Interior of home dark | BCDARK00 | Observer | rowmeans and then Z scored | NA |
| MCS | Stimulating Parenting | House/flat reasonably clean | BCRCLE00 | Observer | rowmeans and then Z scored | NA |
| MCS | Stimulating Parenting | House/flat reasonably uncluttered | BCUNCL00 | Observer | rowmeans and then Z scored | NA |
| MCS | Stimulating Parenting | Parent provided toys during visit | BCTOYS00 | Observer | rowmeans and then Z scored | NA |
| MCS | Stimulating Parenting | Read to (caregiver) | BPOFRE00 | Parent | Princals used to create total Z score | NA |
| MCS | Stimulating Parenting | Read to (anyone) | BPREOF00 | Parent | Princals used to create total Z score | NA |
| MCS | Stimulating Parenting | Going to the library | BPTOLI00 | Parent | Removed due to low loading | NA |
| MCS | Stimulating Parenting | Learn sports | BPSDPA00 | Parent | Removed due to low loading | NA |
| MCS | Stimulating Parenting | Painting and drawing | BPPAMA00 | Parent | Princals used to create total Z score | NA |
| MCS | Stimulating Parenting | Painting and drawing binary | BPDRAW00 | Parent | As there are binary items and ordinal items that can be merged to make 1 better ordinal item. E.g. if someone said they did not do something, they should then get a 0 for the frequency on the next item which indicates how often they do it, rather than a NA which they have in the raw data | NA |
| MCS | Stimulating Parenting | Alphabet (anyone) | BPALPH00 | Parent | As there are binary items and ordinal items that can be merged to make 1 better ordinal item. E.g. if someone said they did not do something, they should then get a 0 for the frequency on the next item which indicates how often they do it, rather than a NA which they have in the raw data | NA |
| MCS | Stimulating Parenting | Frequency being taught letters | BPOFAB00 | Parent | Princals used to create total Z score | NA |
| MCS | Stimulating Parenting | Frequency being taught numbers | BPOFCO00 | Parent | Princals used to create total Z score | NA |
| MCS | Stimulating Parenting | Frequency songs/poems/rhymes | BPOFSO00 | Parent | Princals used to create total Z score | NA |
| MCS | Stimulating Parenting | Teach child songs (anyone) | BPSONG00 | Parent | As there are binary items and ordinal items that can be merged to make 1 better ordinal item. E.g. if someone said they did not do something, they should then get a 0 for the frequency on the next item which indicates how often they do it, rather than a NA which they have in the raw data | NA |
| MCS | Stimulating Parenting | Anyone else reads to child | BPREEL00 | Parent | As there are binary items and ordinal items that can be merged to make 1 better ordinal item. E.g. if someone said they did not do something, they should then get a 0 for the frequency on the next item which indicates how often they do it, rather than a NA which they have in the raw data | NA |
| MCS | Stimulating Parenting | Anyone at home teach child counting | BPNUMB00 | Parent | As there are binary items and ordinal items that can be merged to make 1 better ordinal item. E.g. if someone said they did not do something, they should then get a 0 for the frequency on the next item which indicates how often they do it, rather than a NA which they have in the raw data | NA |
| NLSY79 | Stimulating Parenting | Interviewer: Mother provided toys or interesting activities for child? | CSOB-7A | Observer | rowmeans and then Z scored | NA |
| NLSY79 | Stimulating Parenting | Interviewer: Child's play environment is safe? | CSOB-9A | Observer | rowmeans and then Z scored | NA |
| NLSY79 | Stimulating Parenting | Interviewer: Interior of the home is dark or perceptually monotonous? | CSOB-10B | Observer | rowmeans and then Z scored | NA |
| NLSY79 | Stimulating Parenting | Interviewer: All visible rooms of house/apartment are reasonably clean? | CSOB-11B | Observer | rowmeans and then Z scored | NA |
| NLSY79 | Stimulating Parenting | Interviewer: All visible rooms of house/apartment are minimally cluttered? | CSOB-12B | Observer | rowmeans and then Z scored | NA |
| NLSY79 | Stimulating Parenting | How often does child have a chance to get out of the house? | HOME-A01 | Parent | rowmeans and then Z scored | NA |
| NLSY79 | Stimulating Parenting | About how many children's books does child have? | HOME-A02 | Parent | rowmeans and then Z scored | NA |
| NLSY79 | Stimulating Parenting | How often do you get a chance to read to child? | HOME-A03 | Parent | rowmeans and then Z scored | NA |
| NLSY79 | Stimulating Parenting | How often do you take child to the grocery store? | HOME-A04 | Parent | rowmeans and then Z scored | NA |
| NLSY79 | Stimulating Parenting | About how many, if any, cuddly, soft, or role-playing toys does child have? | HOME-A05 | Parent | rowmeans and then Z scored | NA |
| NLSY79 | Stimulating Parenting | About how many, if any, push or pull toys does child have? | HOME-A06 | Parent | rowmeans and then Z scored | NA |
| NLSY79 | Stimulating Parenting | Some Parents spend time teaching their children new skill while other Parents believe children learn best on their own. Which most closely describes your attitude? | HOME-A07 | Parent | rowmeans and then Z scored | NA |
| NLSY79 | Stimulating Parenting | How many magazines? | MS-HOME-B03 | Parent | rowmeans and then Z scored | NA |
| NLSY79 | Stimulating Parenting | Have tapes or CDs? | MS-HOME-B04 | Parent | rowmeans and then Z scored | NA |
| NLSY79 | Stimulating Parenting | Learn numbers | MS-HOME-B05A | Parent | rowmeans and then Z scored | NA |
| NLSY79 | Stimulating Parenting | Learn alphabet | MS-HOME-B05B | Parent | rowmeans and then Z scored | NA |
| NLSY79 | Stimulating Parenting | Learn colours | MS-HOME-B05C | Parent | rowmeans and then Z scored | NA |
| NLSY79 | Stimulating Parenting | Learn shapes | MS-HOME-B05D | Parent | rowmeans and then Z scored | NA |
| NLSY79 | Stimulating Parenting | Often taken on outing | MS-HOME-B09 | Parent | rowmeans and then Z scored | NA |
| NLSY79 | Stimulating Parenting | Often taken to museum | MS-HOME-B10 | Parent | rowmeans and then Z scored | NA |
| GUI | Temperament | The Fussy /Difficult subscale from the ICQ, consisting of 6 items | fussy | Parent | Z scored | NA |
| GUI | Temperament | Soothability | NA | Parent | NA | NA |
| GUI | Temperament | How often fussy | NA | Parent | NA | NA |
| GUI | Temperament | How much crying in general | NA | Parent | NA | NA |
| GUI | Temperament | Intensity of protest | NA | Parent | NA | NA |
| GUI | Temperament | How changeable is Baby's mood | NA | Parent | NA | NA |
| GUI | Temperament | Overall degree of difficulty | NA | Parent | NA | NA |
| LSAC | Temperament | Irritability scale | airrit | Parent | rowmeans and then Z scored | NA |
| LSAC | Temperament | Approach scale | aaprcha | Parent | rowmeans and then Z scored | NA |
| LSAC | Temperament | Cooperativeness scale | acoop | Parent | rowmeans and then Z scored | NA |
| LSAC | Temperament | Pleasant arrival in new places | ase01a1 | Parent | NA | NA |
| LSAC | Temperament | Still during grooming procedures | ase01b1 | Parent | NA | NA |
| LSAC | Temperament | Happy when changed/dressed | ase01b2 | Parent | NA | NA |
| LSAC | Temperament | Fretful on waking/or going to sleep | ase01c1 | Parent | NA | NA |
| LSAC | Temperament | Accepts strangers at home | ase01a2 | Parent | NA | NA |
| LSAC | Temperament | Accepts grooming without protest | ase01b3 | Parent | NA | NA |
| LSAC | Temperament | Amuses self for 1/2 an hour or more | ase01c2 | Parent | NA | NA |
| LSAC | Temperament | Accepts changes when bathing | ase01a3 | Parent | NA | NA |
| LSAC | Temperament | Distracted from fretting when grooming | ase01b4 | Parent | NA | NA |
| LSAC | Temperament | Continues to cry despite soothing | ase01c3 | Parent | NA | NA |
| LSAC | Temperament | Accepts health care professionals | ase01a4 | Parent | NA | NA |
| LSAC | Temperament | Cries when left to play alone | ase01c4 | Parent | NA | NA |
| MCS | Temperament | Happy sounds during nappy changing etc | APHAPN00 | Parent | Total Score (rowmeans) is used to create a total temperament score where higher score=easier temperament | NA |
| MCS | Temperament | Pleasant first arriving in unfamiliar places. | APUNFA00 | Parent | Total Score (rowmeans) is used to create a total temperament score where higher score=easier temperament | NA |
| MCS | Temperament | Pleasant during hair brushing etc | APBRUS00 | Parent | Total Score (rowmeans) is used to create a total temperament score where higher score=easier temperament | NA |
| MCS | Temperament | Content during interruptions of feeding. | APFEED00 | Parent | Total Score (rowmeans) is used to create a total temperament score where higher score=easier temperament | NA |
| MCS | Temperament | Pleasant or calm with minor injuries | APINJU00 | Parent | Total Score (rowmeans) is used to create a total temperament score where higher score=easier temperament | NA |
| MCS | Temperament | Objects to bathing - different place/person | APBATH00 | Parent | Total Score (rowmeans) is used to create a total temperament score where higher score=easier temperament | NA |
| MCS | Temperament | Wary of strangers after 15 minutes. | APWARY00 | Parent | Total Score (rowmeans) is used to create a total temperament score where higher score=easier temperament | NA |
| MCS | Temperament | Shy on first meeting another child | APBSHY00 | Parent | Total Score (rowmeans) is used to create a total temperament score where higher score=easier temperament | NA |
| MCS | Temperament | Fretful in a new place or situation | APFRET00 | Parent | Total Score (rowmeans) is used to create a total temperament score where higher score=easier temperament | NA |
| MCS | Temperament | Bothered at first by different sleeping place. | APSLEE00 | Parent | Total Score (rowmeans) is used to create a total temperament score where higher score=easier temperament | NA |
| MCS | Temperament | Milk feeds at about the same time | APMILK00 | Parent | Total Score (rowmeans) is used to create a total temperament score where higher score=easier temperament | NA |
| MCS | Temperament | Sleepy at about the same time each evening | APSLTI00 | Parent | Total Score (rowmeans) is used to create a total temperament score where higher score=easier temperament | NA |
| MCS | Temperament | Naps about the same length | APNAPS00 | Parent | Total Score (rowmeans) is used to create a total temperament score where higher score=easier temperament | NA |
| MCS | Temperament | Solid food at about the same time | APSOFO00 | Parent | Total Score (rowmeans) is used to create a total temperament score where higher score=easier temperament | NA |
| NLSY79 | Temperament | Temperament: Difficult Raw Score from the 14 items | DIFFIC | Parent | Z scored | NA |
| NLSY79 | Temperament | Child sleepiness regularity | NA | Parent | total score and then Z scored | NA |
| NLSY79 | Temperament | Child hunger regularity | NA | Parent | total score and then Z scored | NA |
| NLSY79 | Temperament | Child wakes up- mood regularity | NA | Parent | total score and then Z scored | NA |
| NLSY79 | Temperament | Child often afraid of stranger | NA | Parent | total score and then Z scored | NA |
| NLSY79 | Temperament | Child often afraid of animal | NA | Parent | total score and then Z scored | NA |
| NLSY79 | Temperament | Child upset alone | NA | Parent | total score and then Z scored | NA |
| NLSY79 | Temperament | Child often afraid of doctor, dentist or nurse | NA | Parent | total score and then Z scored | NA |
| NLSY79 | Temperament | When you play with child, how often smile or laugh? | NA | Parent | total score and then Z scored | NA |
| NLSY79 | Temperament | When plays alone, how often smile or laugh? | NA | Parent | total score and then Z scored | NA |
| NLSY79 | Temperament | When in the bath, how often smile or laugh? | NA | Parent | total score and then Z scored | NA |
| NLSY79 | Temperament | When child hears an unexpected loud sound, how often cry or become upset? | NA | Parent | total score and then Z scored | NA |
| NLSY79 | Temperament | How often trouble soothing or calming when is crying or upset? | NA | Parent | total score and then Z scored | NA |
| NLSY79 | Temperament | Average day, how often fussy and irritable? | NA | Parent | total score and then Z scored | NA |
| NLSY79 | Temperament | Compared with most babies, how often cry and fuss? | NA | Parent | total score and then Z scored | NA |

# **Appendix S1- Further Description of the Competitive Confirmatory Approach**

The competitive confirmatory approach performs model comparisons by systematically varying the number of parameters needing to be estimated and restricting the values of certain parameters included in a multiple regression equation, with each specification representing a different model of the person-environment interplay. For example, a strict interpretation of the diathesis-stress model says the non-risk characteristic group (easier temperament/higher birthweight) are unaffected by environmental factors and the crossover point between groups is when the environment is high in quality (Figure 1). Thus, the beta coefficient for the main effect of the environment (traditionally beta_1_) is restricted to zero, the characteristic*environmental factor interaction (traditionally beta_3_) is allowed to be freely estimated, and the crossover is fixed to when the environment is highest is quality (Jolicoeur-Martineau et al., 2020). For differential-susceptibility, the strict interpretation is that the main beta effect of the environment is again equal to zero but crucially the interaction should be dis-ordinal (Widaman et al., 2012). A dis-ordinal interaction indicates that the susceptible group have lower performance than the non-susceptible group when both are in low quality parenting environments but that there is a crossover once the environmental quality is higher, within the observable environmental range (Belsky et al., 2013). Finally, strict vantage-sensitivity suggests that those who are vantage sensitive to the environment benefit from more positive environments than vantage resistant individuals, allowing them to have superior developmental outcomes but only once in positive environments. Thus, the vantage resistant group are unaffected by environmental factors (beta_1_=0), the characteristic*environmental factor interaction (beta_3_) is allowed to be freely estimated, and the crossover point is instead fixed to being when the environment is low in quality. To perform these analyses, the LEGIT package in R was used (Jolicoeur-Martineau et al., 2020). The optimal model is then decided by comparing the AIC values for each specification and when applicable, if the crossover point was within the observable environmental range.

**Appendix S2- Further Description of the Proportion Affected (PA) and Proportion of the Interaction(POI) Metrics by Roisman (2012)**

After running a linear mixed model, one can use the metrics proposed in by Roisman (2012) to evaluate whether a model indicates evidence for a specific type of interaction (e.g. diathesis stress or differential susceptibility). After finding a significant interaction term, one can look at whether a crossover occurs between those high (1SD) or low (-1SD) on a certain characteristic (e.g. infant temperament) within the environmental range- i.e. what is shown in a simple slopes figure. For example, if the crossover occurs when the environment is average (Z=0), then in 50% of cases having a difficult infant temperament is advantageous and in 50% of cases it is disadvantageous. This would be evidence for differential susceptibility and would result in a PA of 50% and a POI of 0.50 (assuming the environmental range is perfectly normally distributed). If the crossover is either not within the environmental range, or at an extreme level, then one has evidence for either diathesis stress or vantage sensitivity. In the current analyses, diathesis stress would be indicated by POI and PA values under 0.2 and 16% respectively while vantage sensitivity would be indicated by POI and PA values over 0.8 and 84% respectively. For differential susceptibility, one would therefore need a POI value within the ranges of 0.2 and 0.8 and a PA value within the ranges of 16% and 84%.

For more in depth explantions, see the following descriptions by Fraley on the website <https://www.yourpersonality.net/interaction/>

“Proportion of the Interaction (PoI). The PoI provides a way to express the proportion of the total interaction that is represented on the right side of the crossover point for the interaction. In differential susceptibility theory, this represents the area for which the effect of X on Y is 'for better' if, in fact, a positive association represents a gain rather than a cost. In a prototypical differential susceptibility account, this value will be 50%. In a prototypical diathesis-stress account, this value will be 0% or 100%, depending on the variables in question and whether they represent potential risks or gains.

Proportion Affected (PA). The PA value provides a way to express the proportion of cases that are differentially affected by X. In differential susceptibility theory, this represents the proportion of individuals for whom the effect of X on Y is 'for better'. In a prototypical differential susceptibility account, this value should include a non-trivial proportion of people (e.g., 50%). In a prototypical diathesis-stress account, this value will be closer to 0% (or 100%, depending on how the variables are coded).”

# **Table S2 Extended Demographics using raw variables from cohorts**

|  | **GUI (Ireland) (N=8703)** | **LSAC (Australia) (N=4316)** | **MCS (UK) (N=13124)** | **NLSY79 (USA) (N=4968)** |
| --- | --- | --- | --- | --- |
| **Internalising raw** |  |  |  |  |
| Mean (SD) | 1.23 (1.20) | 1.39 (1.21) | 1.25 (1.26) | 522 (275) |
| Median [Min, Max] | 1.00 [0, 7.50] | 1.00 [0, 7.50] | 1.00 [0, 9.00] | 474 [172, 1000] |
| Missing | 1 (0.0%) | 586 (13.6%) | 334 (2.5%) | 172 (3.5%) |
| **Externalising raw** |  |  |  |  |
| Mean (SD) | 2.35 (1.67) | 2.69 (1.63) | 2.37 (1.70) | 505 (276) |
| Median [Min, Max] | 2.00 [0, 9.50] | 2.50 [0, 10.0] | 2.00 [0, 10.0] | 486 [127, 1000] |
| Missing | 1 (0.0%) | 584 (13.5%) | 335 (2.6%) | 172 (3.5%) |
| **Crystal IQ Raw** |  |  |  |  |
| Mean (SD) | 111 (18.1) | 65.3 (6.04) | 107 (19.8) | 88.7 (21.1) |
| Median [Min, Max] | 112 [26.0, 170] | 66.2 [34.2, 84.8] | 107 [10.0, 170] | 91.0 [0, 160] |
| Missing | 109 (1.3%) | 176 (4.1%) | 0 (0%) | 344 (6.9%) |
| **Fluid IQ Raw** |  |  |  |  |
| Mean (SD) | 86.9 (11.6) | 10.7 (2.98) | 81.3 (14.3) | 101 (14.3) |
| Median [Min, Max] | 85.0 [10.0, 119] | 10.0 [3.00, 19.0] | 82.0 [10.0, 119] | 101 [65.0, 135] |
| Missing | 72 (0.8%) | 288 (6.7%) | 0 (0%) | 653 (13.1%) |
| **Birthweight (g)** |  |  |  |  |
| Mean (SD) | 3500 (535) | 3420 (566) | 3350 (594) | 3350 (610) |
| Median [Min, Max] | 3500 [1500, 4600] | 3460 [382, 5440] | 3370 [390, 6780] | 3370 [454, 7600] |
| Missing | 96 (1.1%) | 17 (0.4%) | 8 (0.1%) | 2 (0.0%) |
| **Temperament raw** |  |  |  |  |
| Mean (SD) | 14.8 (4.87) | -13.4 (1.84) | 2.13 (0.431) | 27.4 (7.26) |
| Median [Min, Max] | 14.0 [6.00, 39.0] | -13.5 [-18.0, -6.75] | 2.08 [1.00, 4.09] | 28.0 [11.0, 54.0] |
| Missing | 19 (0.2%) | 492 (11.4%) | 346 (2.6%) | 807 (16.2%) |

# **Table S3 Correlation matrix of all main variables**

|  | Birth  weight | Temperament | Crystal IQ | Fluid IQ | Internalising behaviours | Externalising behaviours | Sensitive Parenting Maternal | Sensitive Parenting Observer | Stimulating Parenting Maternal | Stimulating Parenting Observer |
| --- | --- | --- | --- | --- | --- | --- | --- | --- | --- | --- |
| Birthweight | 1 | . | . | . | . | . | . | . | . | . |
| Temperament | .03 | 1 | . | . | . | . | . | . | . | . |
| Crystal IQ | .08 | .07 | 1 | . | . | . | . | . | . | . |
| Fluid IQ | .07 | .04 | .48 | 1 | . | . | . | . | . | . |
| Internalising behaviours | .06 | .15 | .15 | .14 | 1 | . | . | . | . | . |
| externalising behaviours | .05 | .14 | .16 | .18 | .42 | 1 | . | . | . | . |
| Sensitive Parenting Maternal | .03 | .16 | .12 | .09 | .20 | .32 | 1 | . | . | . |
| Sensitive Parenting Observer | .05 | .09 | .20 | .13 | .13 | .13 | .13 | 1 | . | . |
| Stimulating Parenting Maternal | .01 | .11 | .20 | .12 | .10 | .16 | .21 | .18 | 1 | . |
| Stimulating Parenting Observer | .04 | .08 | .17 | .12 | .14 | .14 | .11 | .32 | .15 | 1 |

# **Table S4 Optimal model consistency across cohorts (4= same optimal model in all 4 cohorts)**

|  | | | | | | | |
| --- | --- | --- | --- | --- | --- | --- | --- |
| *Independent and Dependent Variables* | *Optimal Model* | | | | | | ***Total*** |
|  | No Effects | Environment Main Effect | Individual Characteristic Main Effect | Independent/Additive Effects | Diathesis Stress | Vantage Sensitivity |  |
| Birthweight and Sens-Par-MR on Behaviour-Externalising | 0 | 1 | 0 | 1 | 0 | 2 | 4 |
| Birthweight and Sens-Par-MR on Behaviour-Internalising | 0 | 0 | 0 | 2 | 2 | 0 | 4 |
| Birthweight and Sens-Par-MR on IQ-Crystallised | 0 | 0 | 0 | 3 | 0 | 1 | 4 |
| Birthweight and Sens-Par-MR on IQ-Fluid | 0 | 0 | 0 | 0 | 1 | 3 | 4 |
| Birthweight and Stim-Par-MR on Behaviour-Externalising | 0 | 1 | 0 | 1 | 1 | 1 | 4 |
| Birthweight and Stim-Par-MR on Behaviour-Internalising | 0 | 0 | 0 | 0 | 4 | 0 | 4 |
| Birthweight and Stim-Par-MR on IQ-Crystallised | 0 | 0 | 0 | 1 | 2 | 1 | 4 |
| Birthweight and Stim-Par-MR on IQ-Fluid | 0 | 0 | 0 | 2 | 1 | 1 | 4 |
| Easy-Temp and Sens-Par-MR on Behaviour-Externalising | 0 | 0 | 0 | 3 | 0 | 1 | 4 |
| Easy-Temp and Sens-Par-MR on Behaviour-Internalising | 0 | 0 | 0 | 4 | 0 | 0 | 4 |
| Easy-Temp and Sens-Par-MR on IQ-Crystallised | 0 | 1 | 0 | 0 | 2 | 1 | 4 |
| Easy-Temp and Sens-Par-MR on IQ-Fluid | 0 | 1 | 0 | 1 | 1 | 1 | 4 |
| Easy-Temp and Stim-Par-MR on Behaviour-Externalising | 0 | 0 | 0 | 3 | 0 | 1 | 4 |
| Easy-Temp and Stim-Par-MR on Behaviour-Internalising | 0 | 0 | 0 | 3 | 0 | 1 | 4 |
| Easy-Temp and Stim-Par-MR on IQ-Crystallised | 0 | 0 | 0 | 1 | 1 | 2 | 4 |
| Easy-Temp and Stim-Par-MR on IQ-Fluid | 0 | 3 | 0 | 1 | 0 | 0 | 4 |
| ***Total*** | 0 | 7 | 0 | 26 | 15 | 16 | 64 |
|  | | | | | | | |

# **Appendix S3 Nonlinear Testing**

Please see <https://osf.io/732nx> for the R markdown, interactive version of these figures and table.

**Nonlinear Diathesis Stress/Vantage Sensitivity/Differential Susceptibility Testing**

In this analysis, we wish to test for nonlinear relationships between the independent variables (infant temperament and birthweight) and the dependent variables (IQ and behavioural outcomes). To test this, we make changes to the “main analysis” where we pool data from all cohorts and use maternal reported parenting variables but use the LEGIT R package which tests for linear effects when determining whether there is evidence for additive effects, diathesis stress, vantage sensitivity and differential susceptibility. For these new analyses, we have a total of 16 models based on 2 infant characteristics (birth weight or temperament) x 2 parenting factors (stimulating or sensitive parenting) x 4 outcomes (crystallised IQ, fluid IQ, internalising problems, externalising problems). We want to test each combination for nonlinear effects and then determine which person-environment interaction pattern is best supported. How this is done is explained below,

**The new models tested- all include the test for main effects of infant characteristic, environmental factor and their respective interaction:**

1. multiple regression with both the individual characteristic and environmental (parenting) factor as continuous variables (essentially the same as the original analysis)
2. multiple regression with the individual characteristic as a 3-level categorical variable (low, medium high- based on being above or below 1SD) and the environmental factor still as a continuous variable.
3. multiple regression with both the individual characteristic and environmental factor as continuous variables but testing for a quadratic relationship between the predictors and the outcome (squared relationship)
4. multiple regression with both the individual characteristic and environmental factor as continuous variables but testing for a cubic relationship between the predictors and the outcome

**The 8 possible interaction patterns to test**

After running the four regressions, we determine which regression is optimal based on having the lowest AIC value. From the optimal regression, we will then calculate the estimated marginal means which will then help us to determine whether the interaction patterns indicate a specific type of relationship. There are many possible patterns. Here are the 8 that I will look for:

1. Additive effects (both environment and individual characteristic factors are significant but no significant interaction)
2. Only environmental factor (no significant individual characteristic or interaction term)
3. Only individual characteristic (no significant environmental factor or interaction term)
4. Clear Diathesis Stress (significant interaction, the lowest performing group in poor environments are more affected by the environment factor but there is no crossover with other groups within the environmental range)
5. Clear Vantage Sensitivity (significant interaction, groups do not differ in poor environments but one group is more positively affected by the environment factor. There is no crossover with other groups within the environmental range)
6. Clear Differential Susceptibility (significant interaction, the lowest performing group in poor environments are more affected by the environment factor and there is crossover with other groups within the environmental range)
7. Overlapping Diathesis Stress/Differential Susceptibility (further described below)
8. Overlapping Vantage Sensitivity/Differential Susceptibility (further described below)

**Rules to Determine the Interaction Pattern**

In order to determine which pattern is correct, we will use the following rules/tests, largely based on calculating and contrasting the marginal means, where each pattern is potentially ruled out one by one:

1. When environment Z = -2 (poor environment) we determine which group has the lowest estimated marginal means (usually infants with a difficult infant temperament). When environment Z =2 (good environment), has there been a crossover (i.e. are difficult infants no longer the group with the lowest marginal mean?) - If they are always the lowest then this rules out differential susceptibility as there is no crossover.
2. At any environmental value, is the infant characteristic significantly associated with the outcome? This determines the effect of the individual characteristic and so rules out the environmental only pattern.
3. Is the average slope of the environmental factor significantly different to zero (i.e. regardless of the value of the infant characteristic)? If yes, this rules out the individual characteristic only pattern.
4. Is the difference between the infants with normal and difficult temperaments similar at X=-2 and X=2? If yes, this indicates that there is no infant characteristic*environmental factor interaction and so rules out clear vantage sensitivity and clear diathesis stress
5. Does the difference between the infants with a normal temperament and a difficult temperament get significantly bigger between X=-2 and X=2? If yes, this then indicates vantage sensitivity and so rules out clear diathesis stress
6. Does the difference between the with a normal temperament and a difficult temperament get smaller between X=-2 and X=2? If yes, this then indicates diathesis stress and so rules out clear vantage sensitivity
7. When there is a disordinal interaction, at what value of X does the lowest infant group at X =-2 first overtake another group? In perfect differential susceptibility this should equal approximately 0 (i.e. perfectly disordinal). If the value equals -1, it indicates Overlapping Vantage Sensitivity/Differential Susceptibility. If the value equals 1, it indicates Overlapping Diathesis Stress/Differential Susceptibility.

**How the Code Works**

The code follows a specific pattern, ruling out possible interaction patterns at each step:

1. Run the 4 regression models (linear, categorical, squared and cubic) and determine which has the lowest AIC
2. Calculate the estimated marginal means and determine if and where the crossover is
3. Determine if there are any significant associations between infant characteristic and outcome
4. Determine if there are any significant associations between parenting factor (the environment) and outcome
5. Determine if the difference between groups of infants stays the same throughout the environmental range
6. Determine if the difference between infants diminishes/grows throughout the environmental range

**Results- IPD Non-Linear Analyses Table**

From the 16 models, here is an overview of what we find regarding the nonlinear effects and the interaction pattern. Surprisingly, the squared or cubic model is almost always optimal based on the AIC value (except for one case where a categorical easy-normal-difficult temperament factor provided a more parsimonious model). We then plot the marginal means from each model in the plots below.

|  | **Model Number** | **Outcome** | **Infant Factor** | **Environmental Factor** | **Model type** | **Model supported** |
| --- | --- | --- | --- | --- | --- | --- |
| 1 | 1 | IQ-Crys | Easy-Temp | Stim-Par-MR | Cubic | Additive Effect, |
| 2 | 2 | IQ-Crys | Easy-Temp | Sens-Par-MR | Categorical | Additive Effect, |
| 3 | 3 | IQ-Crys | Birthweight | Stim-Par-MR | Cubic | Diathesis Stress, |
| 4 | 4 | IQ-Crys | Birthweight | Sens-Par-MR | Squared | Additive Effect, |
| 5 | 5 | IQ-Fluid | Easy-Temp | Stim-Par-MR | Cubic | Additive Effect, |
| 6 | 6 | IQ-Fluid | Easy-Temp | Sens-Par-MR | Cubic | Additive Effect, |
| 7 | 7 | IQ-Fluid | Birthweight | Stim-Par-MR | Squared | Additive Effect, |
| 8 | 8 | IQ-Fluid | Birthweight | Sens-Par-MR | Squared | Vantage Sensitivity, |
| 9 | 9 | Int-Pos | Easy-Temp | Stim-Par-MR | Squared | Additive Effect, |
| 10 | 10 | Int-Pos | Easy-Temp | Sens-Par-MR | Squared | Additive Effect, |
| 11 | 11 | Int-Pos | Birthweight | Stim-Par-MR | Squared | Diathesis Stress, |
| 12 | 12 | Int-Pos | Birthweight | Sens-Par-MR | Squared | Diathesis Stress, |
| 13 | 13 | Ext-Pos | Easy-Temp | Stim-Par-MR | Squared | Vantage Sensitivity, |
| 14 | 14 | Ext-Pos | Easy-Temp | Sens-Par-MR | Cubic | Vantage Sensitivity, |
| 15 | 15 | Ext-Pos | Birthweight | Stim-Par-MR | Squared | Additive Effect, |
| 16 | 16 | Ext-Pos | Birthweight | Sens-Par-MR | Cubic | Additive Effect, |

**Results- IPD Non-Linear Analyses Plots**

In order to understand the 16 plots below, there are a number of acronyms/labels that need to be described/explained. Generally, the plots are like simple slopes where group (birthweight or easier temperament) is split into low (-1) medium (0) or high (1). After calculating the estimated marginal means at 5 different values of parenting (from very poor, X= -2) to very good (x=2), we can determine whether the pattern indicates a specific interaction pattern.

LGC= Lowest Group Consistent. This determines whether the group (e.g. difficult infant temperament have a group value of -1) that was lowest at X=-2 is consistently lower at all other values of X. If the number = 5 then they are always the lowest, a value of 2 or 3 would indicate differential susceptibility as the interaction is disordinal.

Diff_Sta= Difference stable. This tests the difference of the difference. I.e. whether the difference between the normal temp group (group=0) and the other groups when X=-2 is statistically different to the difference when instead X =2. If the difference of the difference is non-significant then it indicates a stable difference over time.

Diff_Grows= Difference Grows. Similar to above but if the difference to the normal group gets significantly bigger as X increase, this indicates vantage sensitivity

Diff_Dim= Difference Diminishes. Similar to above but if the difference to the normal group gets significantly smaller as X increase, this indicates diathesis stress

Env_Eff = Environment effect. Testing whether X (parenting factor) has a significant association with Y (the outcome), rules out the Individual Characteristic only models.

Sig_From_Normal= At a specific value of X, do either of the groups significantly differ to the normal group? For example, a square indicates that the easy infants do not differ from the normal temperament infants at this value of X. In comparison, a circle indicates that the marginal means do differ at this value of X. If any are significantly different to the normal group (Sig_From_Normal=TRUE), this rules out the environment only models.

## Model 1 Plot


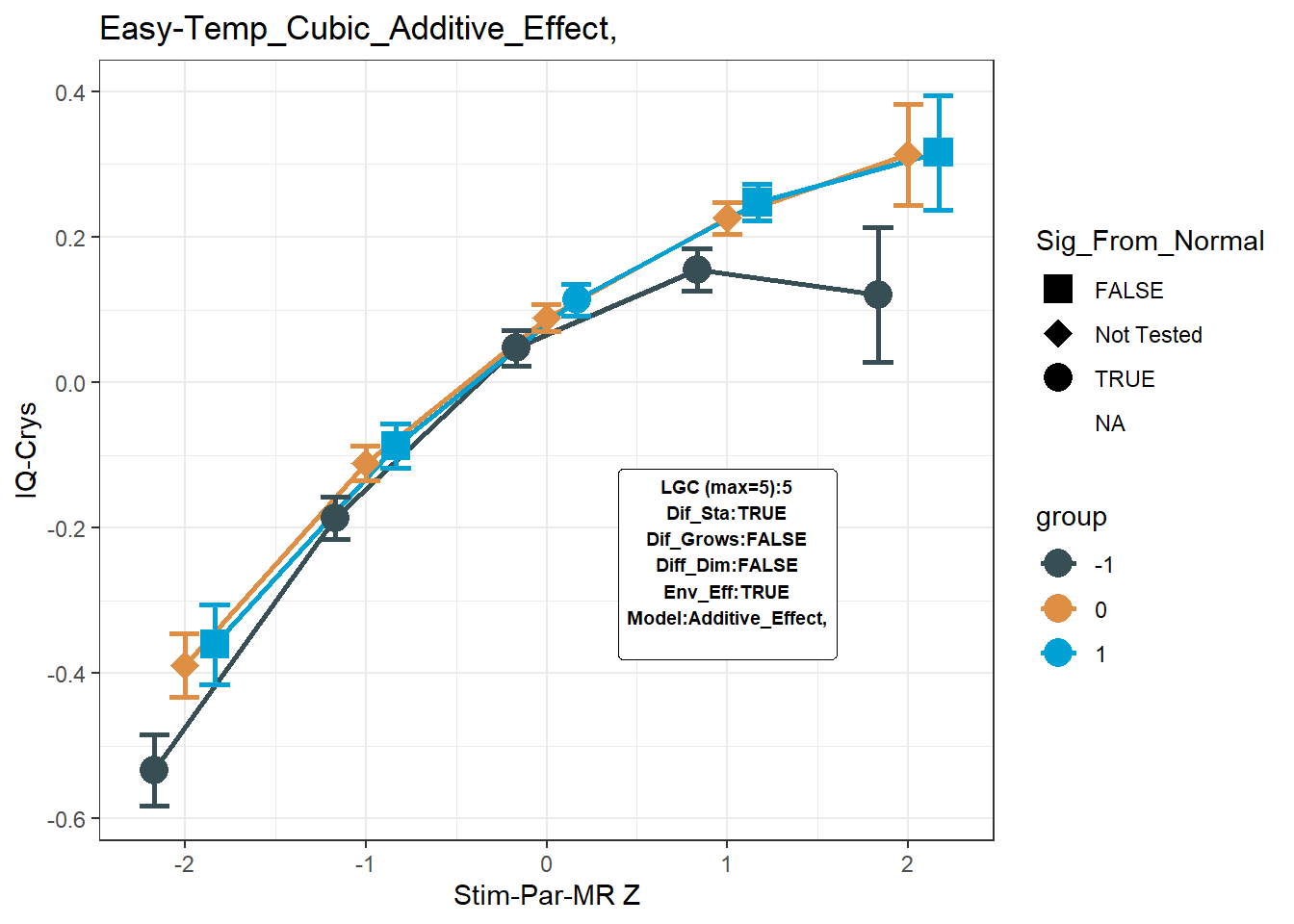


## Model 2 Plot


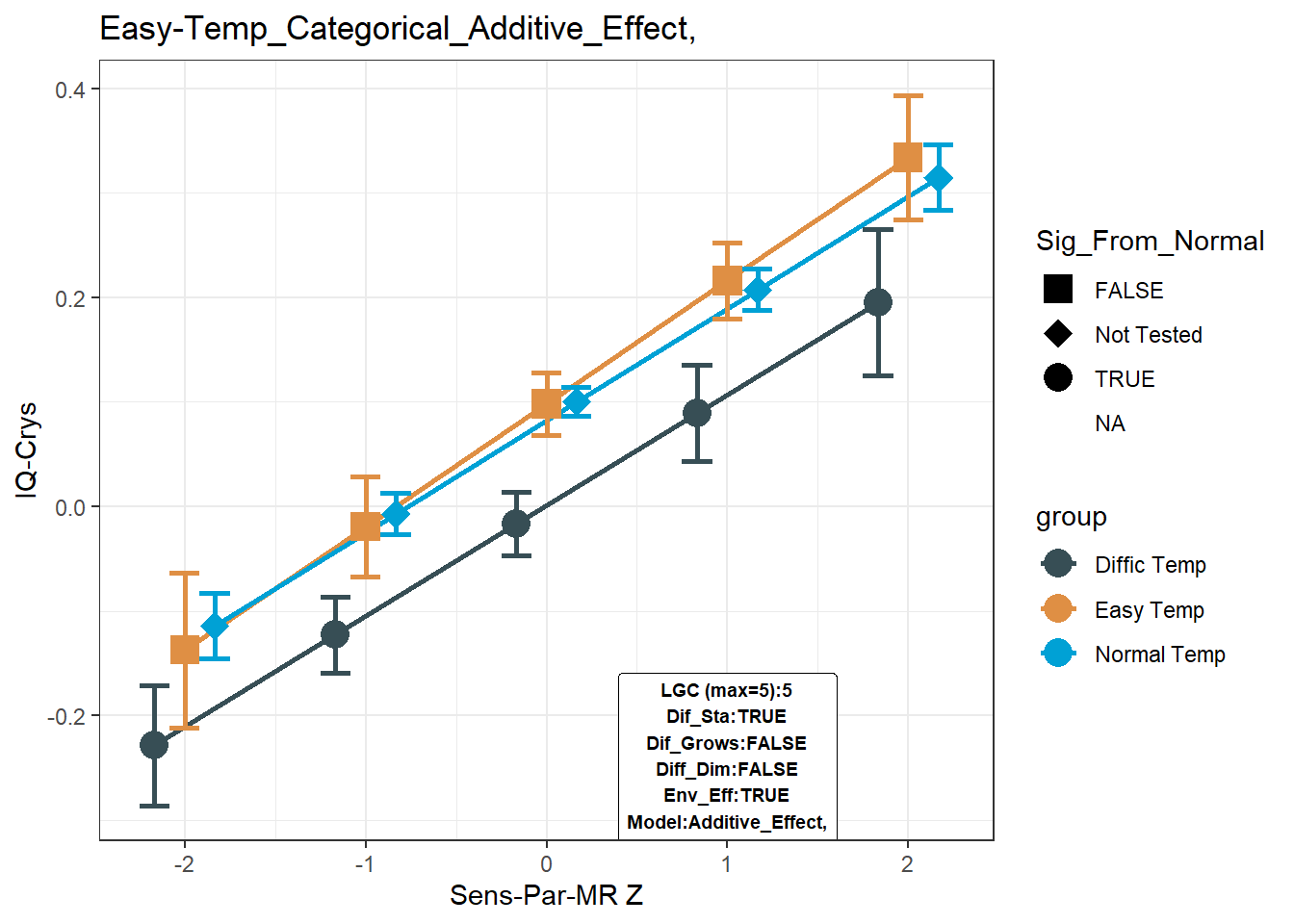


## Model 3 Plot


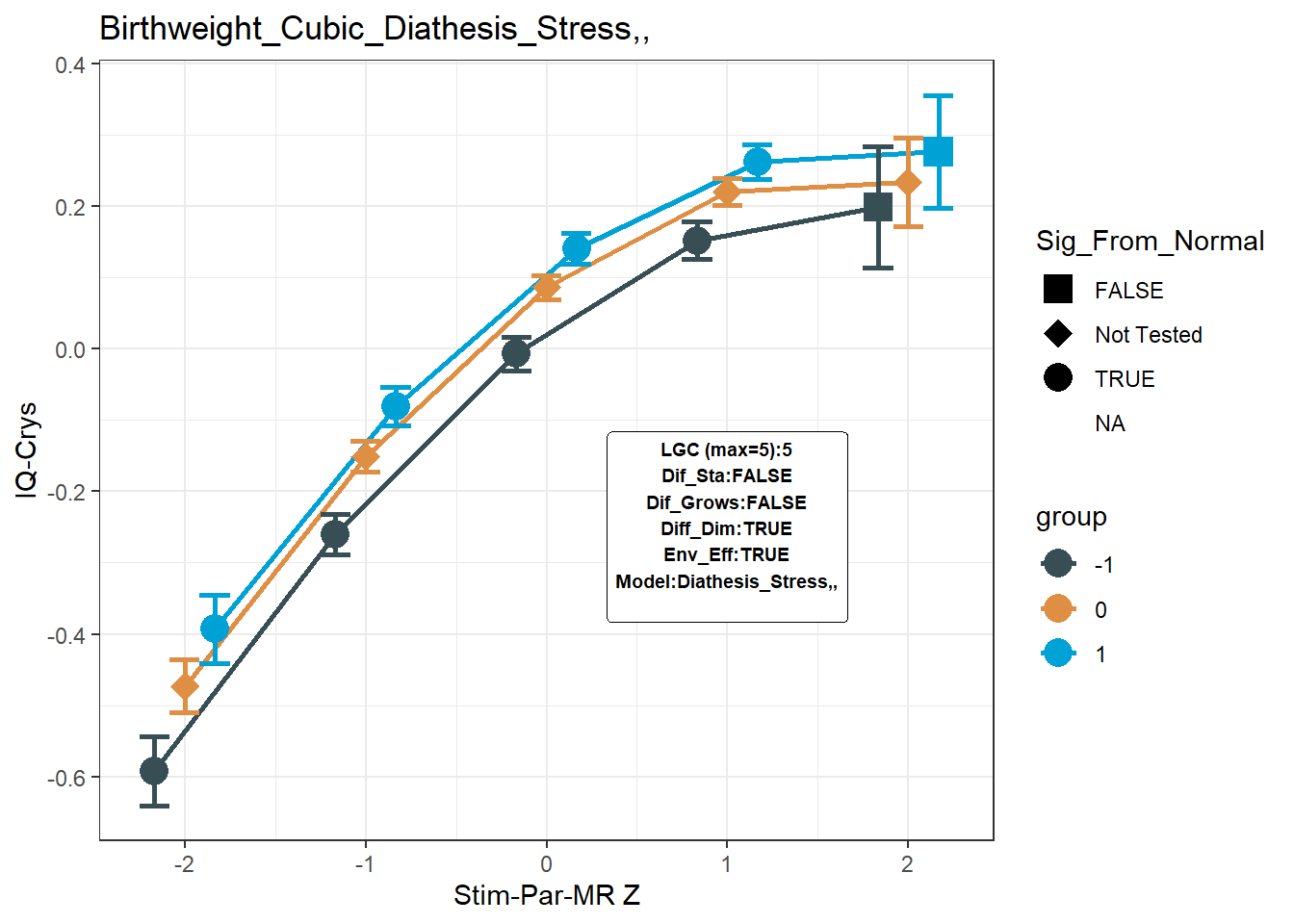


## Model 4 Plot


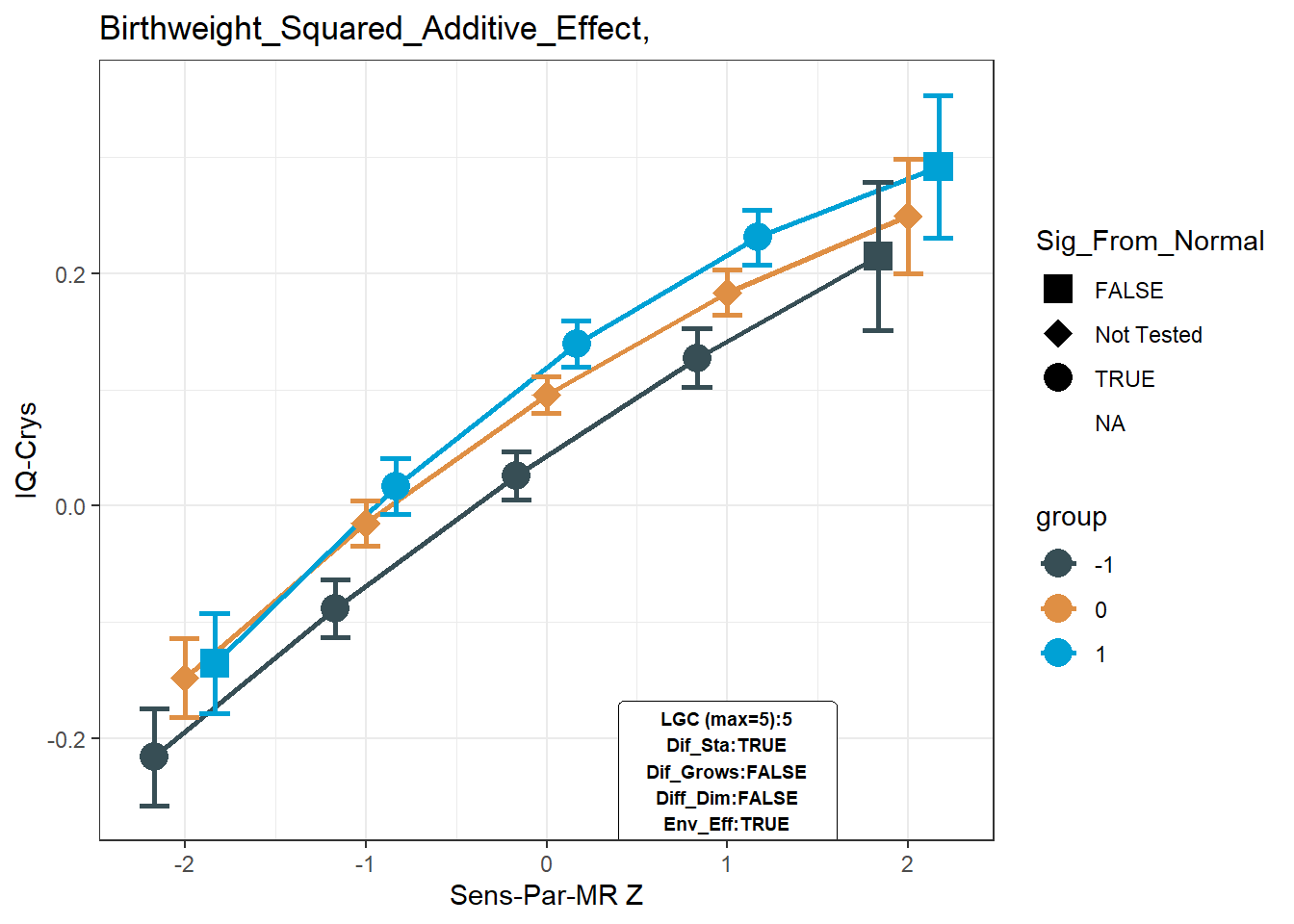


## Model 5 Plot


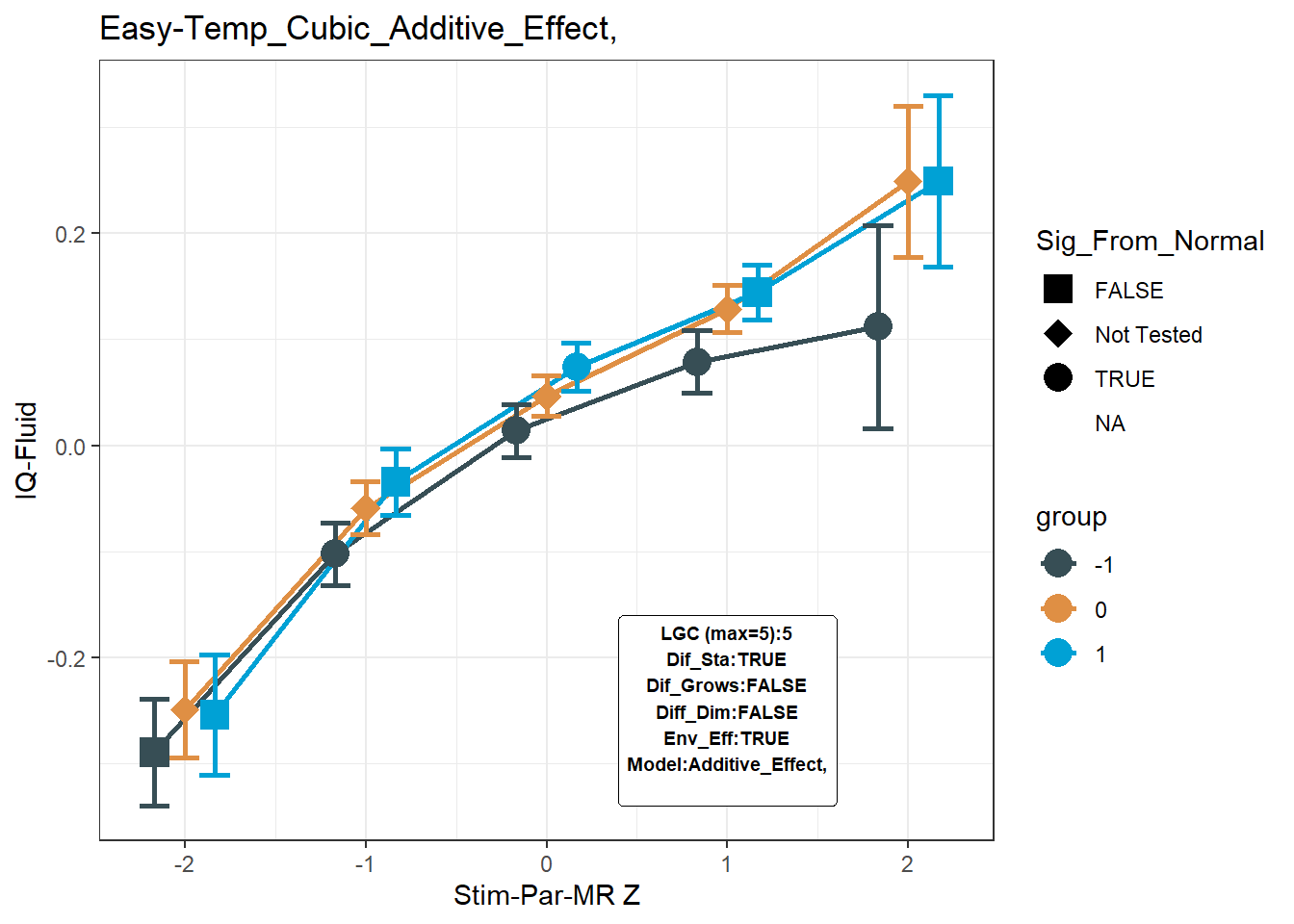


## Model 6 Plot


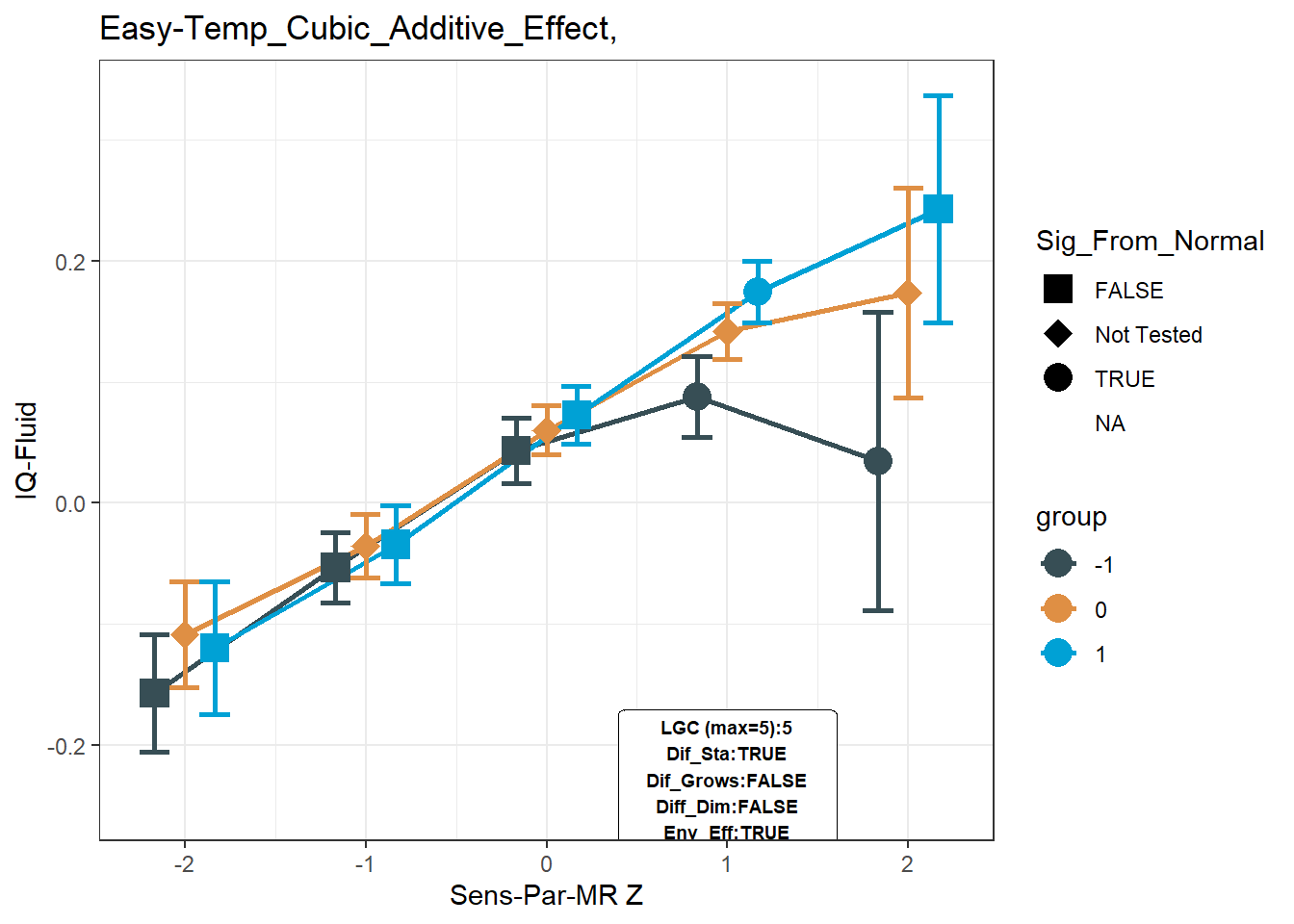


## Model 7 Plot


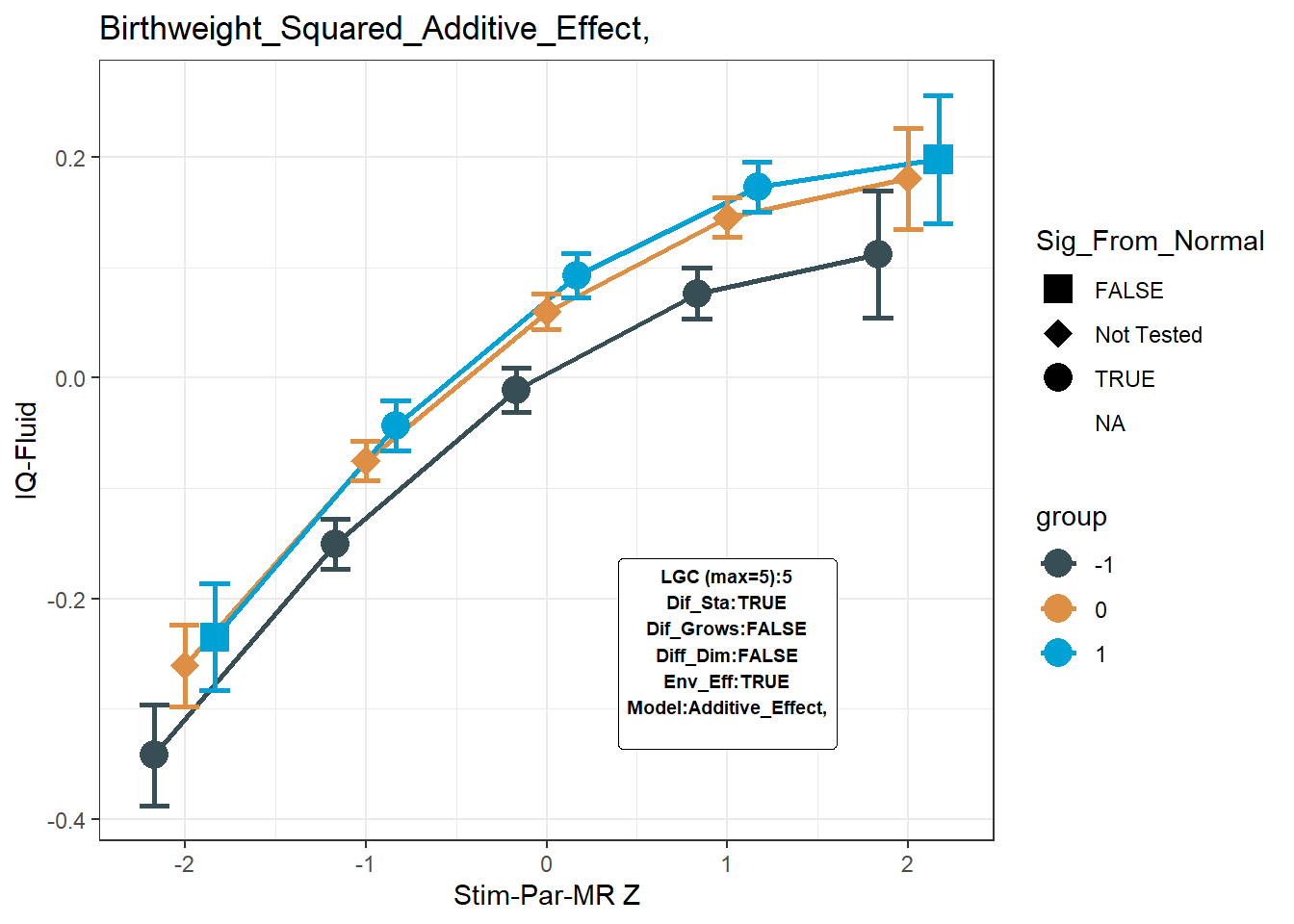


## Model 8 Plot


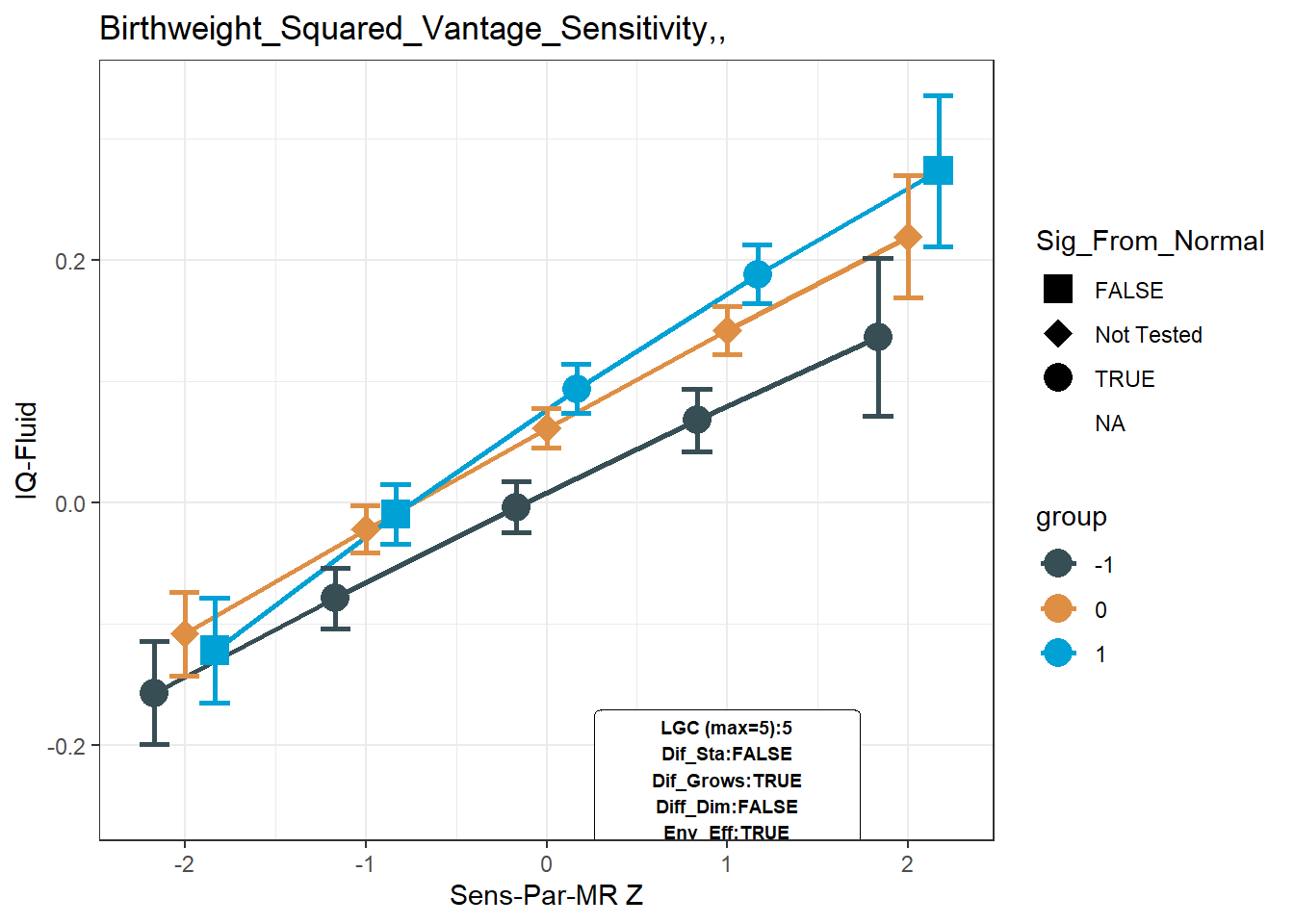


## Model 9 Plot


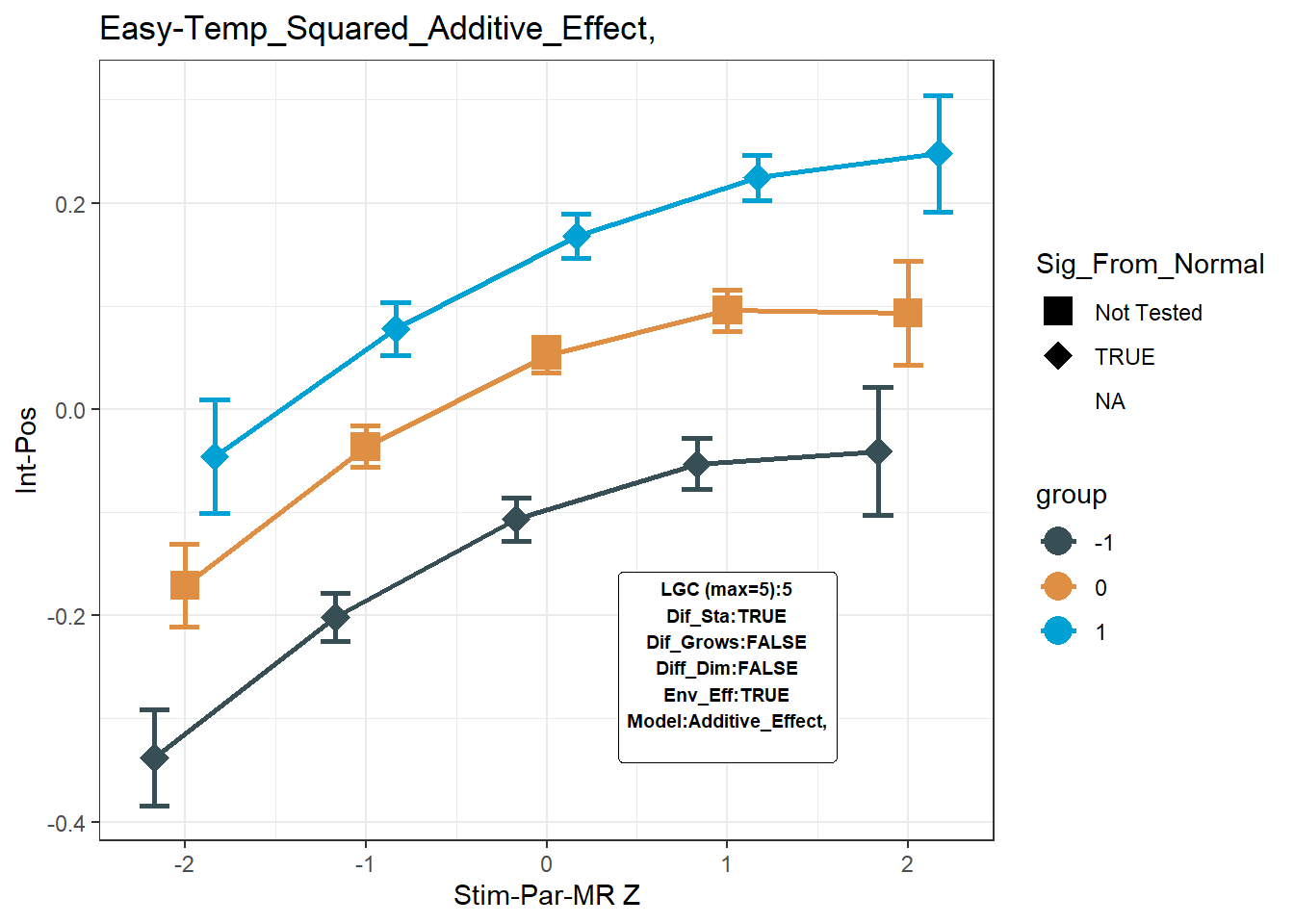


## Model 10 Plot


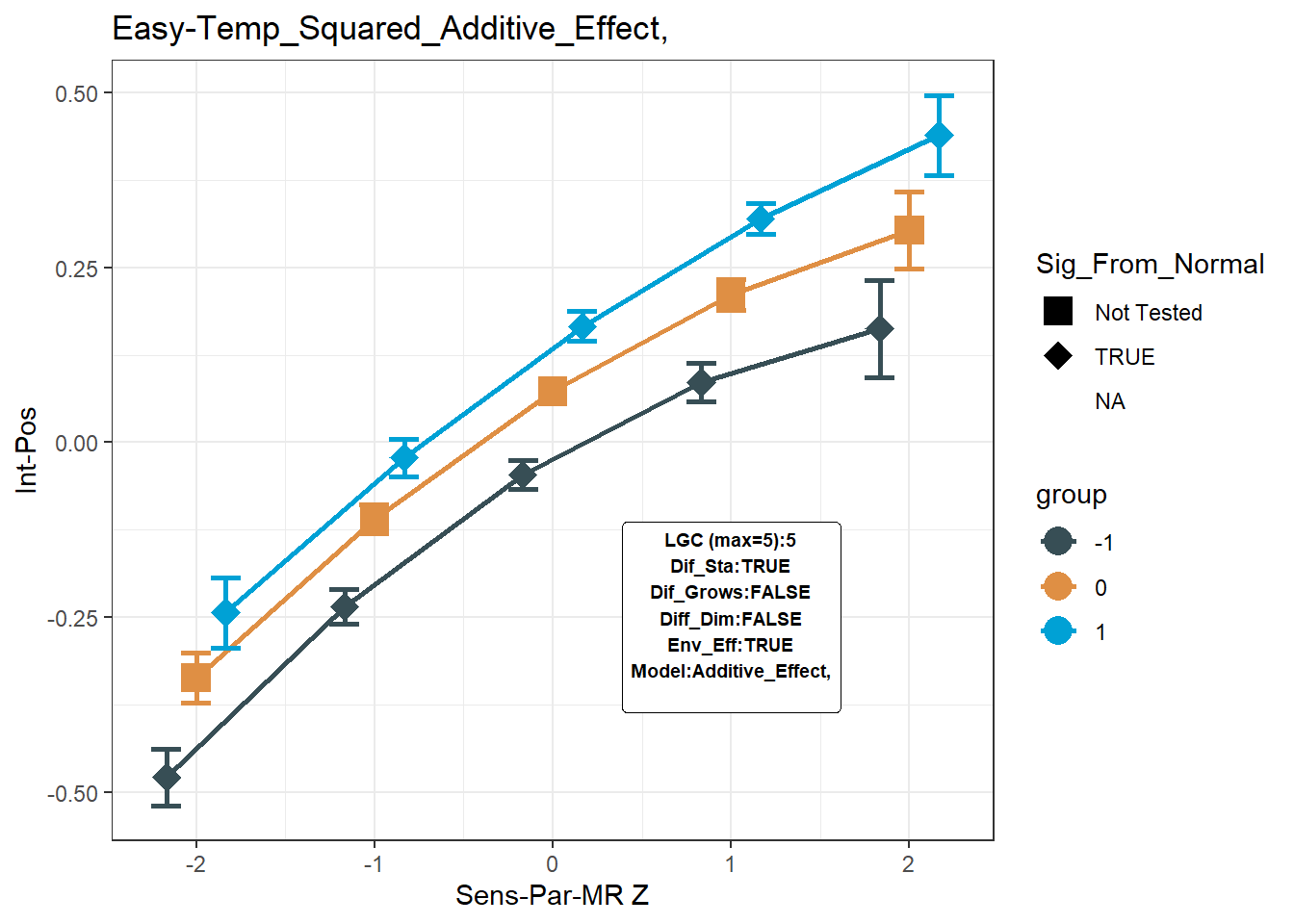


## Model 11 Plot


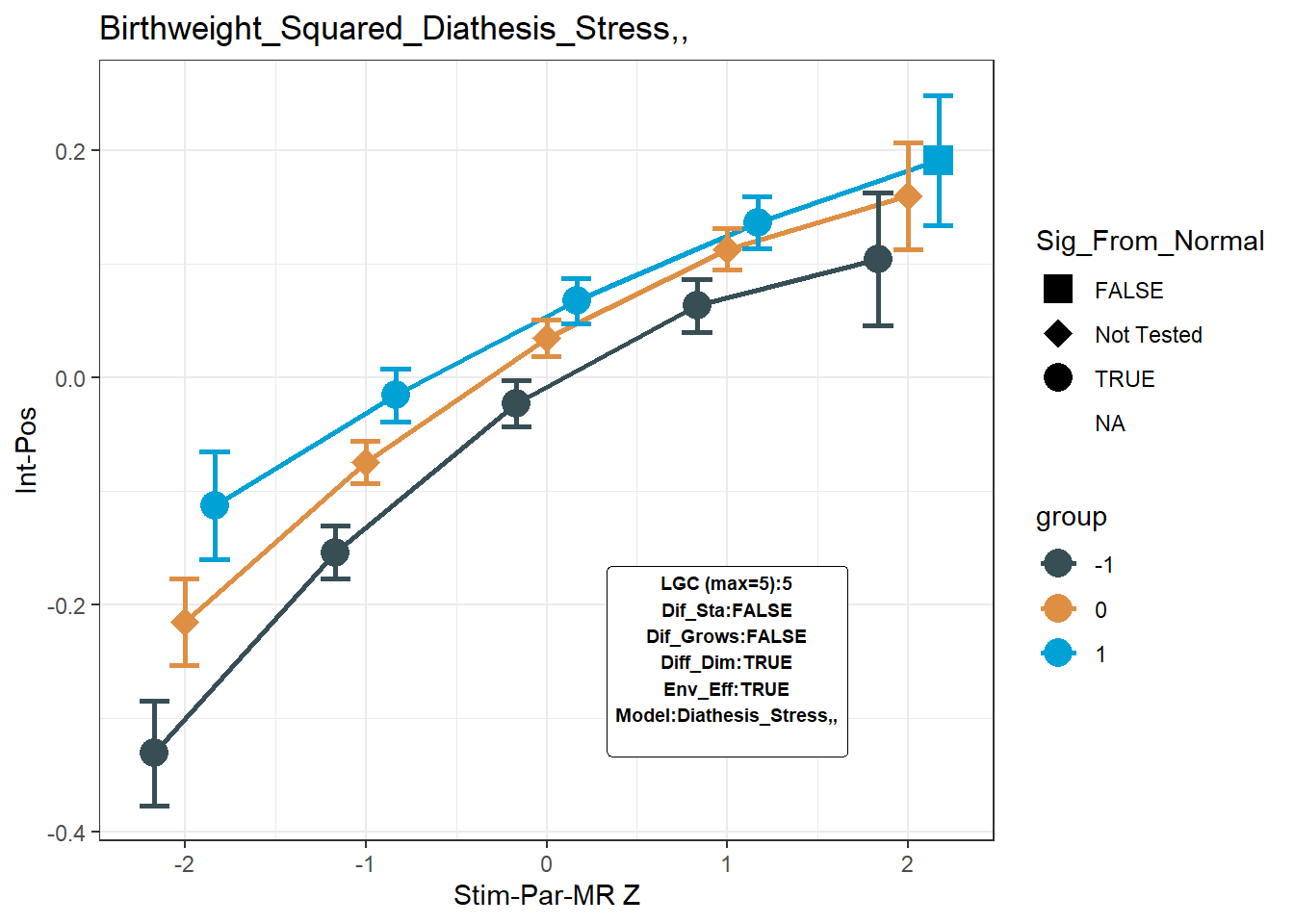


## Model 12 Plot


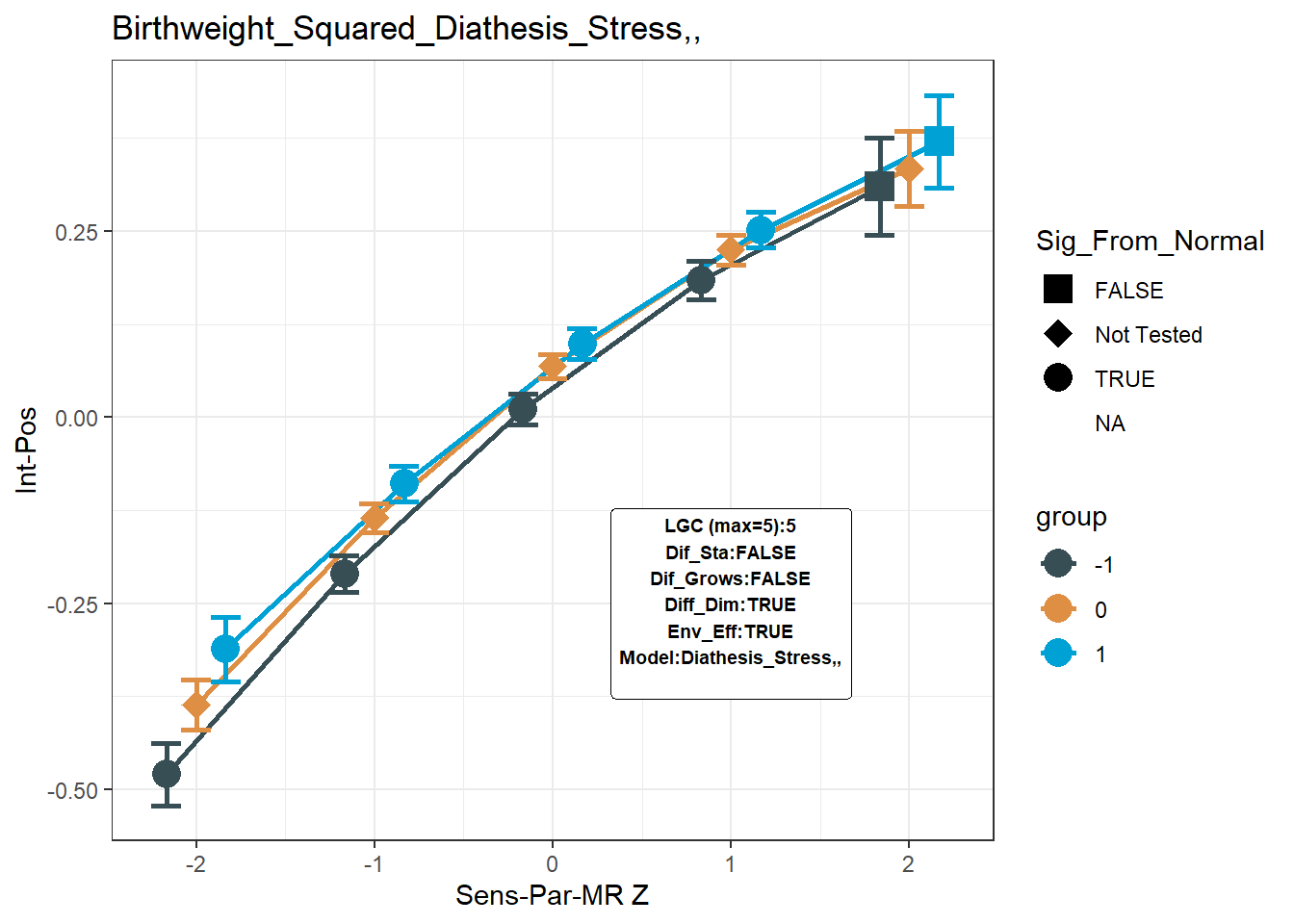


## Model 13 Plot


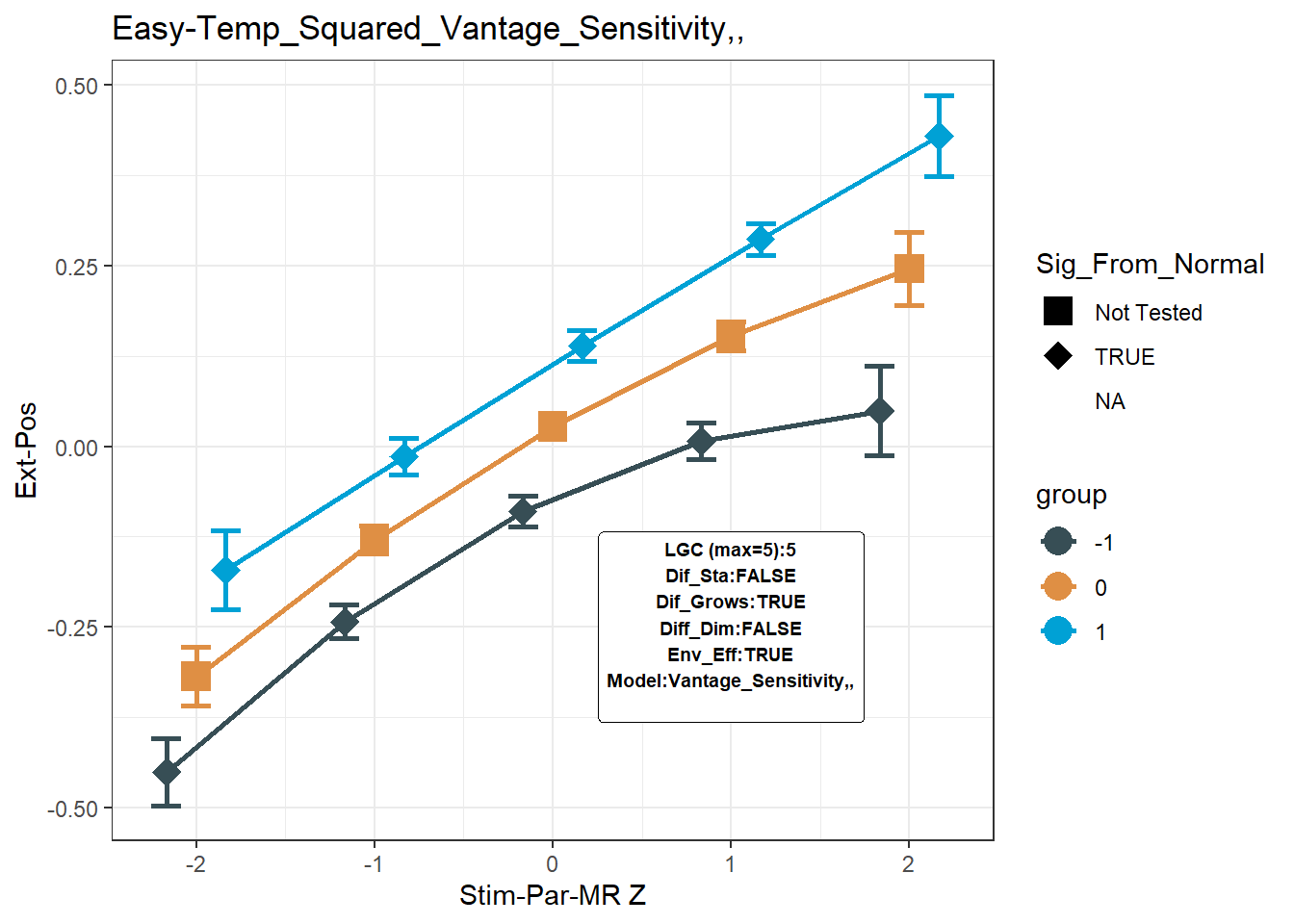


## Model 14 Plot


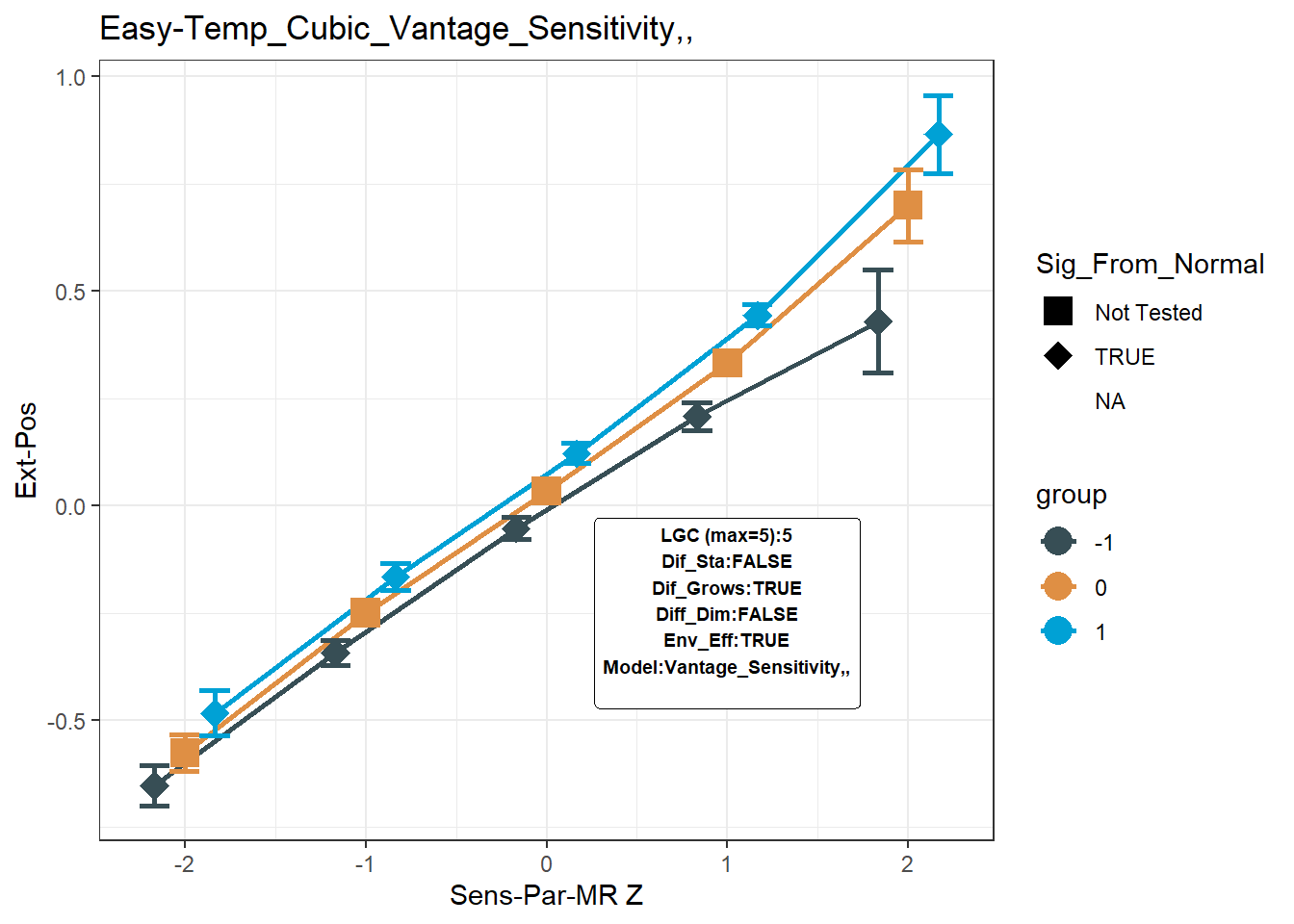


## Model 15 Plot


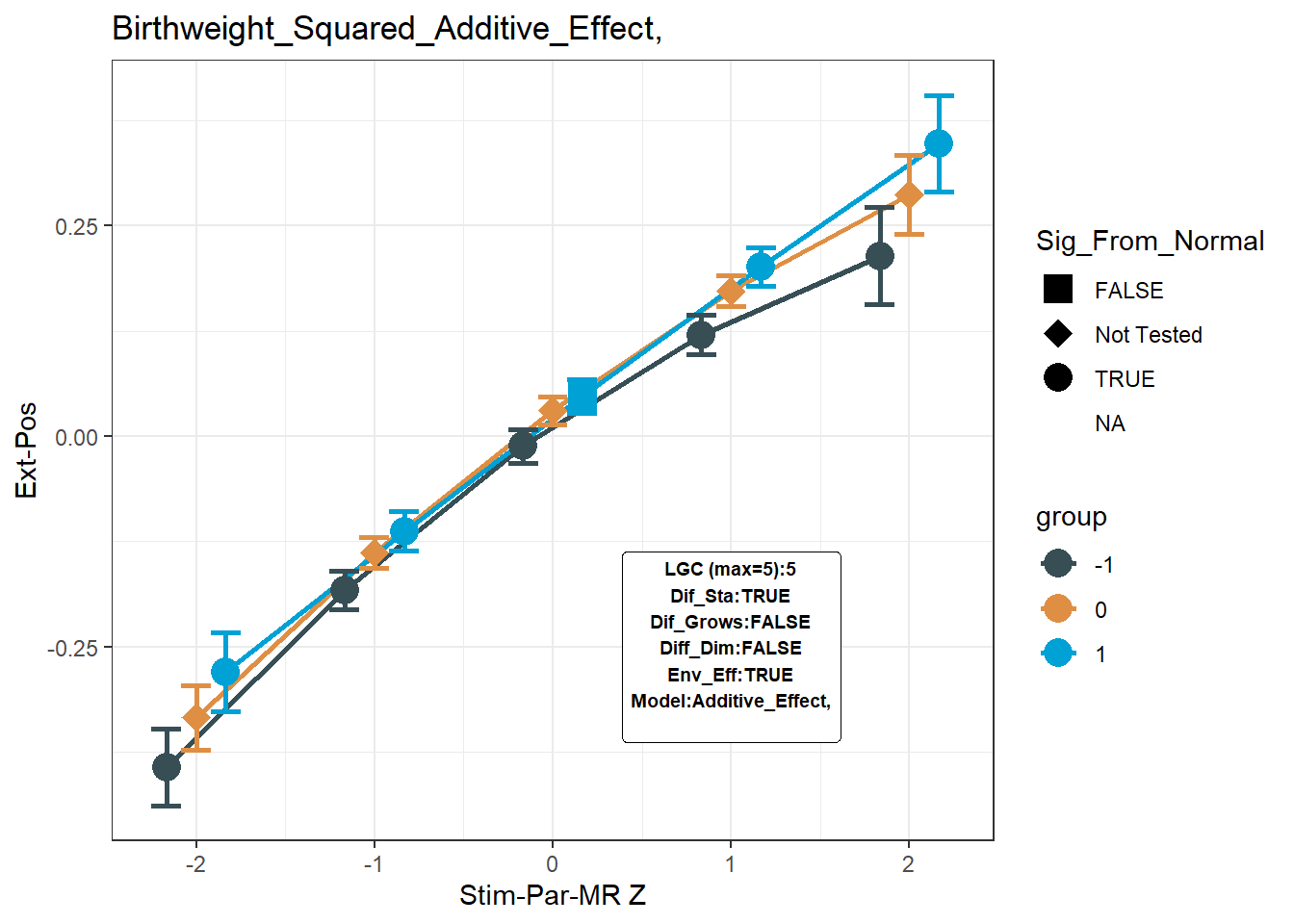


## Model 16 Plot


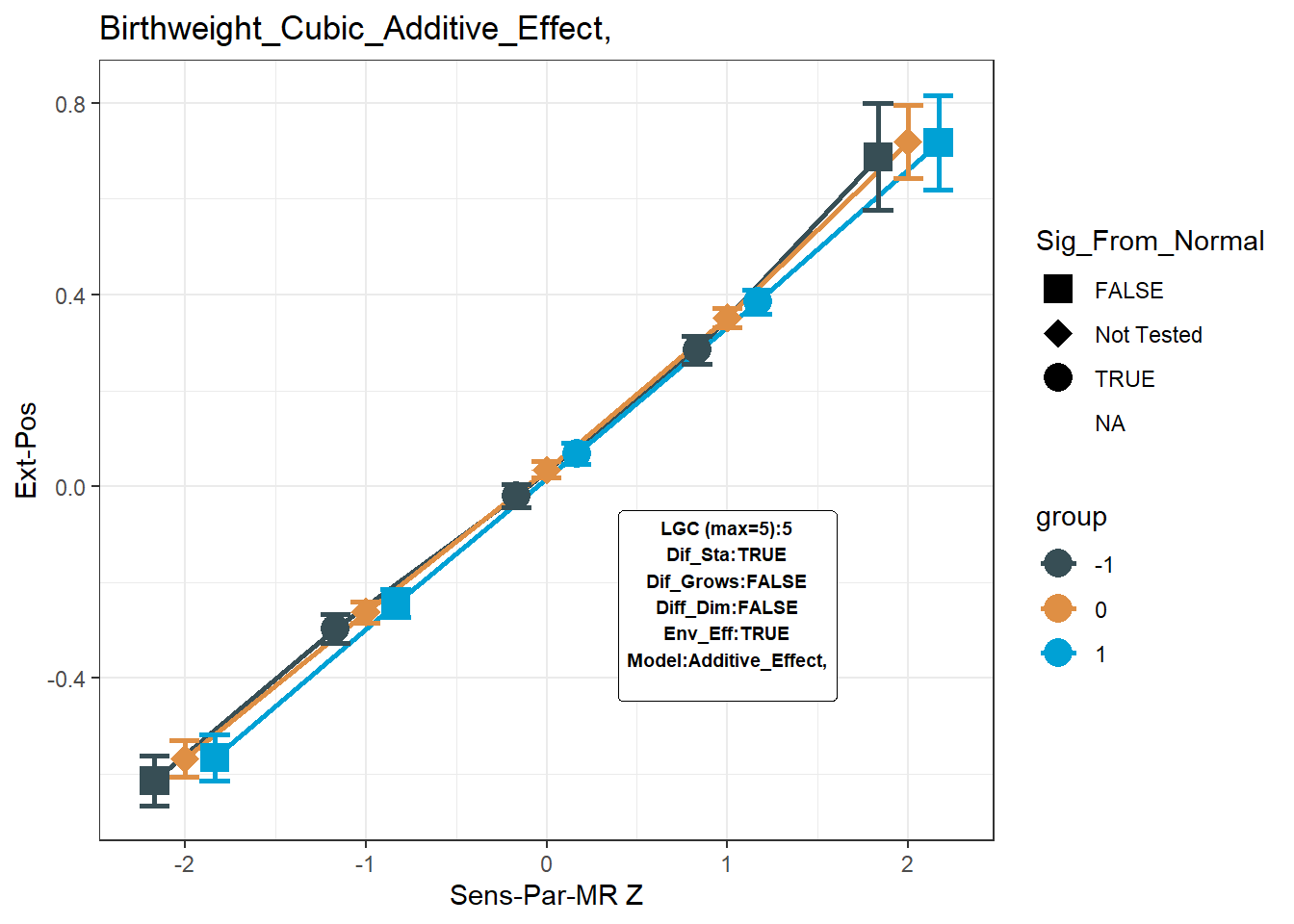


**Figure S1: Johnson-Neyman plots from the 16 IPD:1S analyses**


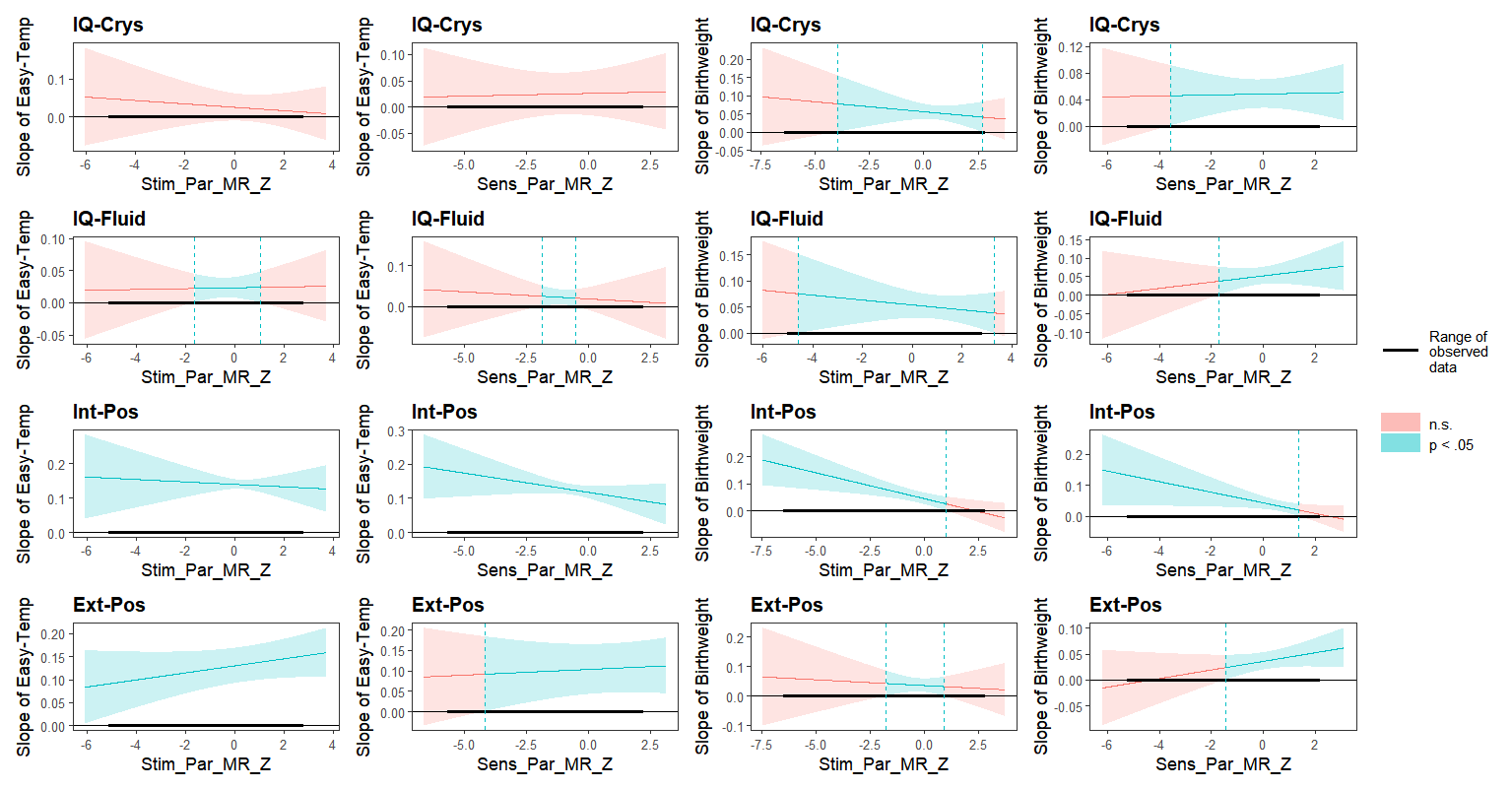


**Figure S2: Simple slope plots from the 16 IPD:1S analyses**


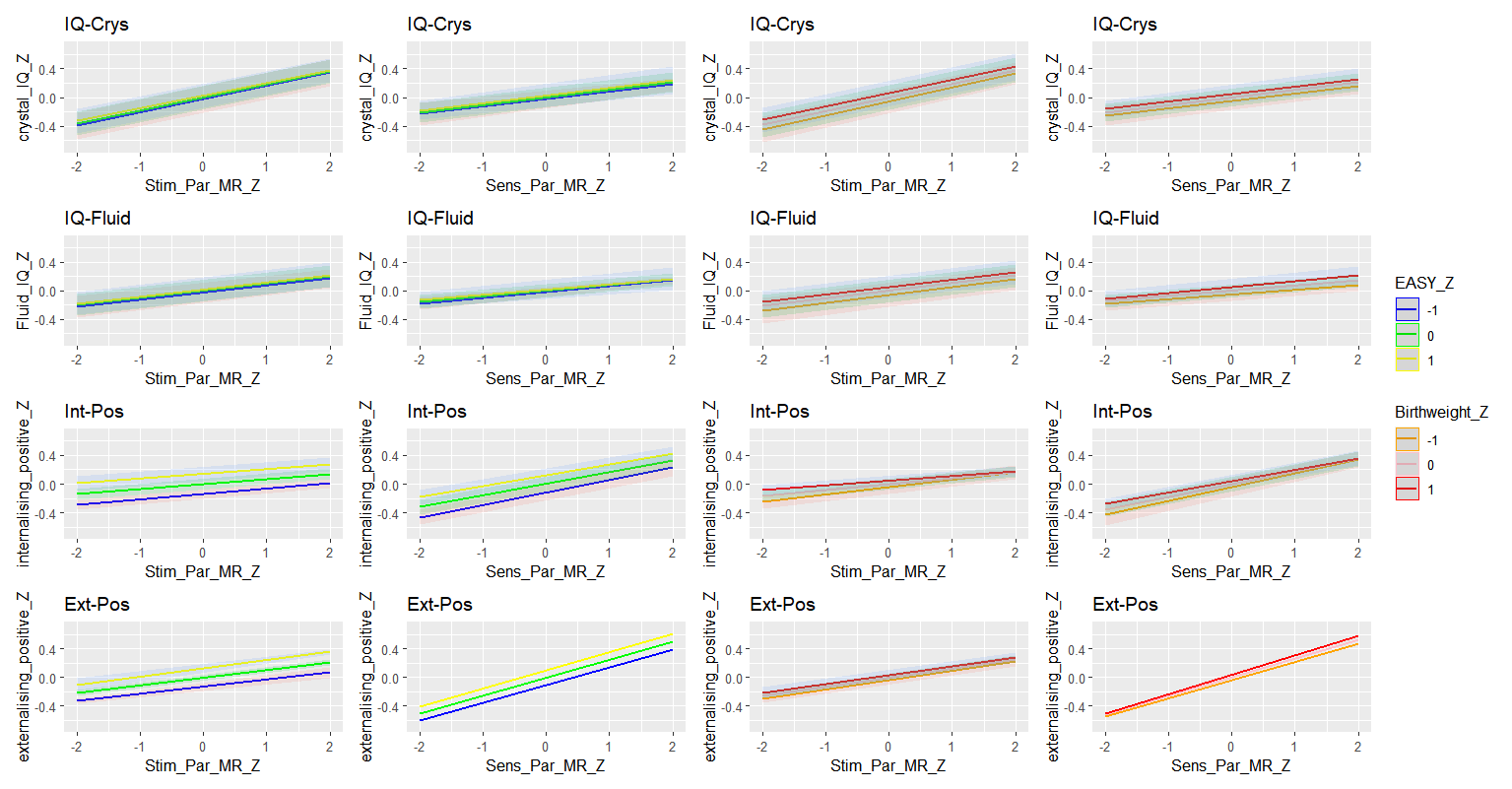


**Figure S3: Infant temperament’s association on developmental outcomes depending on stimulating or sensitive parenting- without covariates**


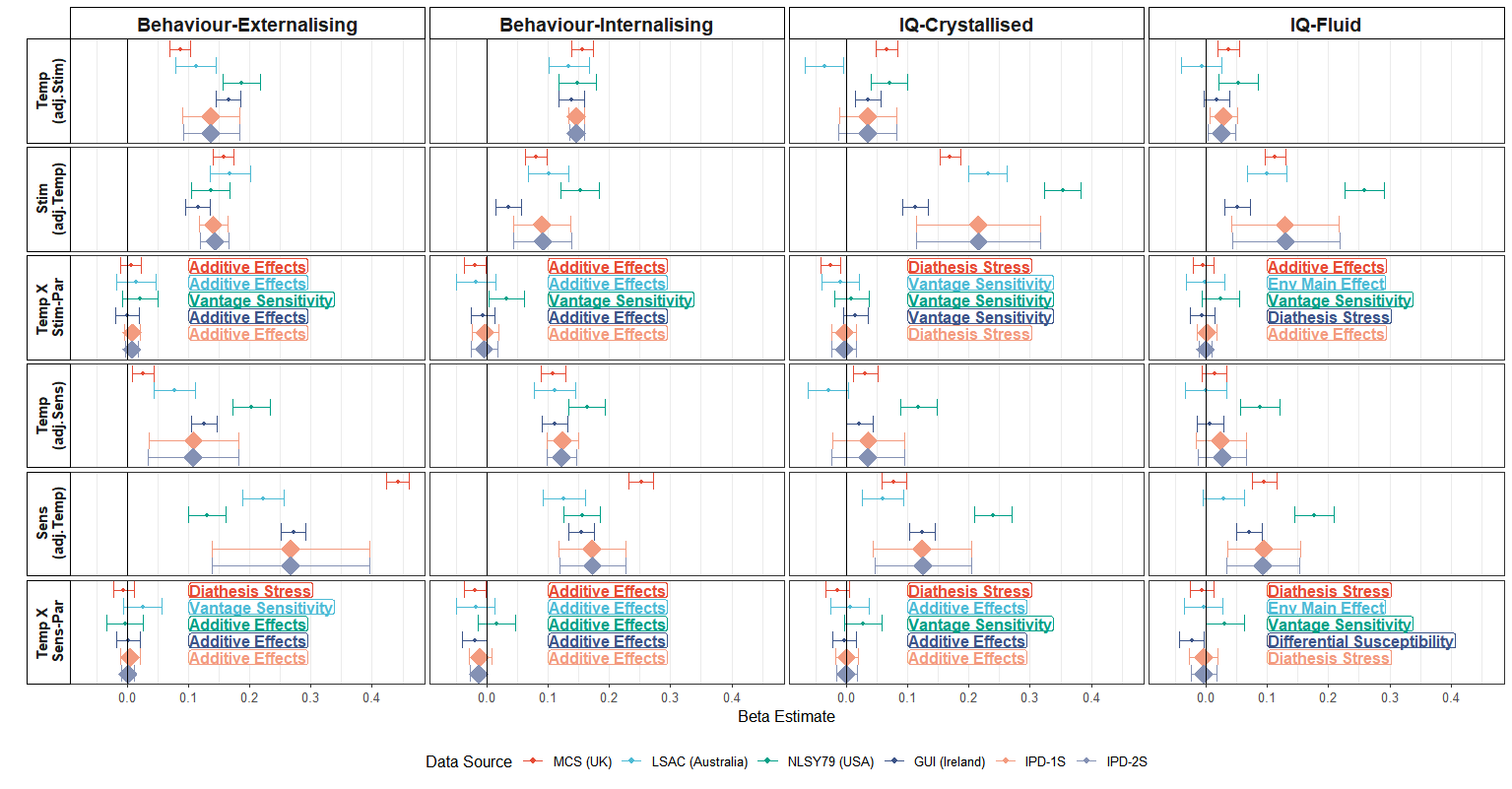


**Figure S4: Birthweight’s association on developmental outcomes depending on stimulating or sensitive parenting- without covariates**

**
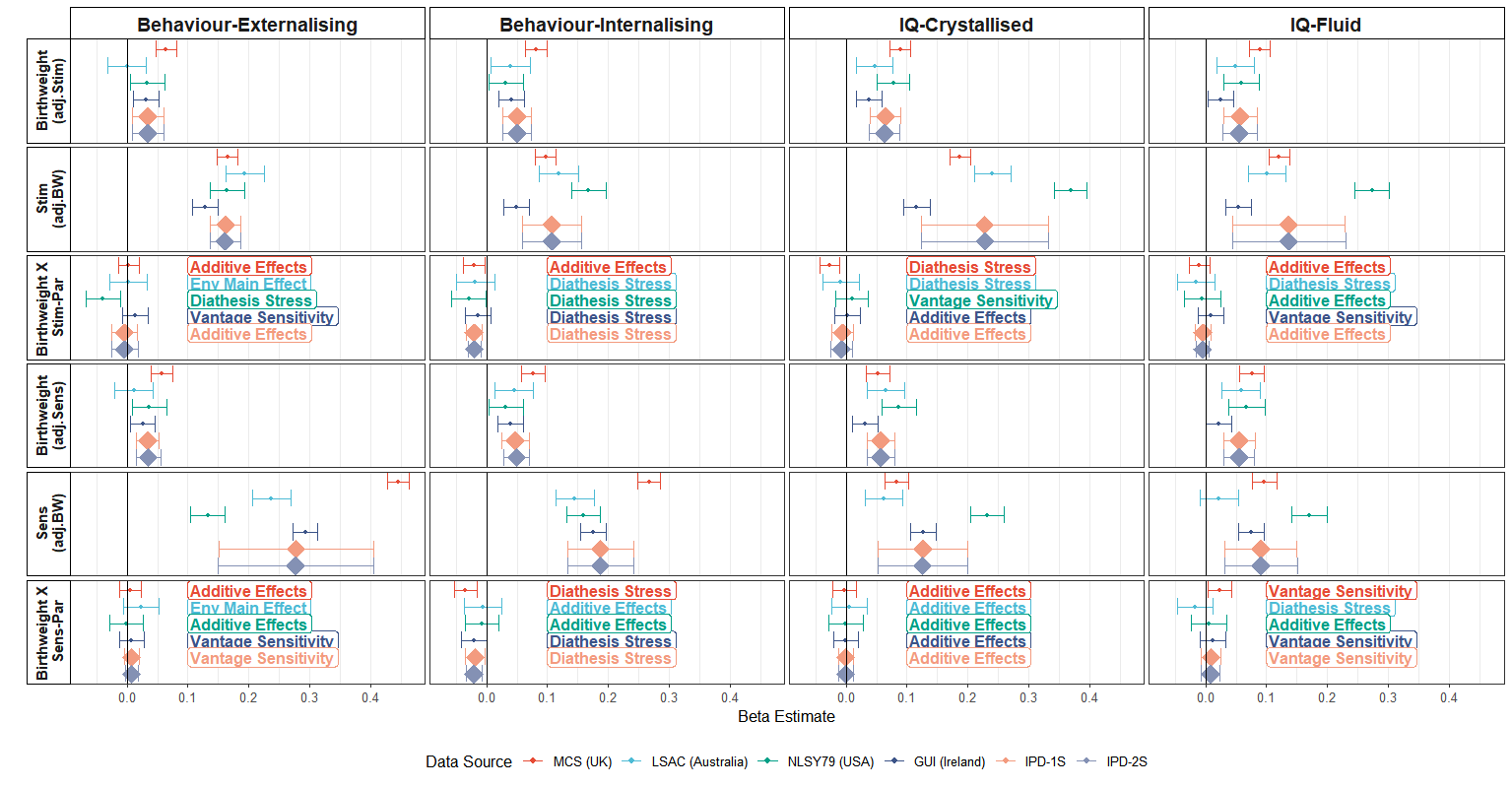
**
